# Neurogenesis in primates versus rodents and the value of non-human primate models

**DOI:** 10.1093/nsr/nwad248

**Published:** 2023-09-15

**Authors:** Runrui Zhang, Hongxin Quan, Yinfeng Wang, Fucheng Luo

**Affiliations:** State Key Laboratory of Primate Biomedical Research; Institute of Primate Translational Medicine, Kunming University of Science and Technology, Kunming 650500, China; Yunnan Key Laboratory of Primate Biomedical Research, Kunming 650500, China; State Key Laboratory of Primate Biomedical Research; Institute of Primate Translational Medicine, Kunming University of Science and Technology, Kunming 650500, China; Yunnan Key Laboratory of Primate Biomedical Research, Kunming 650500, China; State Key Laboratory of Primate Biomedical Research; Institute of Primate Translational Medicine, Kunming University of Science and Technology, Kunming 650500, China; Yunnan Key Laboratory of Primate Biomedical Research, Kunming 650500, China; State Key Laboratory of Primate Biomedical Research; Institute of Primate Translational Medicine, Kunming University of Science and Technology, Kunming 650500, China; Yunnan Key Laboratory of Primate Biomedical Research, Kunming 650500, China

**Keywords:** rodent, primate, embryonic neurogenesis, adult neurogenesis

## Abstract

Neurogenesis, the process of generating neurons from neural stem cells, occurs during both embryonic and adult stages, with each stage possessing distinct characteristics. Dysfunction in either stage can disrupt normal neural development, impair cognitive functions, and lead to various neurological disorders. Recent technological advancements in single-cell multiomics and gene-editing have facilitated investigations into primate neurogenesis. Here, we provide a comprehensive overview of neurogenesis across rodents, non-human primates, and humans, covering embryonic development to adulthood and focusing on the conservation and diversity among species. While non-human primates, especially monkeys, serve as valuable models with closer neural resemblance to humans, we highlight the potential impacts and limitations of non-human primate models on both physiological and pathological neurogenesis research.

## INTRODUCTION

Neurogenesis, the process of generating neurons from neural stem cells that contribute to brain development and cognitive functions, has always been of interest to the field of neuroscience. Neural stem cells originate from neuroepithelial (NE) cells of the neural tube during embryonic development. With the transition of NE cells into radial glial (RG) cells, neural stem cells start to differentiate and give rise to neurons that mark the beginning of neurogenesis. Neurogenesis is very active during development and becomes restricted in adulthood. While our understanding of human neurogenesis and brain development has significantly advanced in recent years (reviewed in [1,2]), numerous questions remain due to ethical and material limitations. The intricacies of neurogenesis during embryonic development and adulthood pose challenges for direct investigation. Ethical considerations restrict the accessibility of human embryonic samples, impeding comprehensive studies of early neurodevelopment. Moreover, studying neurogenesis in the adult human brain is constrained by limited sample availability and the complex interplay of various factors that affect neuronal production.

Recent advancements have highlighted the importance of studying non-human primates to gain deeper insights into human neurogenesis and its related disorders. Non-human primates, such as monkeys and apes, share significant anatomical and functional similarities with humans, making them valuable models for understanding the complex processes of neurogenesis and primate species-specificity. In comparison to rodents, primates undergo an expansion of cortex during their embryonic development, which involves a slower NE to RG transition [3] and the presence of a notable region called the outer subventricular zone (OSVZ) enriched with outer radial glia (oRG) cells [4–6]. During embryonic neurogenesis, these oRG cells possess greater proliferation and division capacity and can generate a wider range of neuron types and quantities, which cause the evolution of the cerebral cortex from a lissencephalic state to a gyrencephalic state [7,8]. Conversely, neurogenesis after birth in primates declines during childhood, with levels dropping further in adulthood and becoming restricted to specific niches, such as the subventricular zone (SVZ) of the brain ventricle and the subgranular zone (SGZ) of the hippocampal gyrus. Especially in adult human brains, hippocampal neurogenesis and its function have been a topic of debate for a long time and requires deeper investigation in primates [9–18]. In addition, disruptions in neurogenesis are associated with various neurological disorders, including neurodevelopmental diseases in the embryonic stage and neurodegenerative diseases and mood disorders in the adult stage [19]. Insights gained from studying neurogenesis in non-human primates under physiological and pathological conditions extend our understanding of human neurodevelopment and hold promise for neurological disorder therapies.

Considering the above reasons, we provide a review mainly focusing on neurogenesis in non-human primates and humans. In this review, we summarize embryonic and adult neurogenesis in rodents and primates, highlighting their similarities and differences. We also discuss the impacts of neurogenesis on neurological disorders, aiming to foster a deeper understanding of neurogenesis and its implications for therapeutic interventions.

## EMBRYONIC NEUROGENESIS IN PRIMATES VERSUS RODENTS

During early embryonic development, the process of gastrulation results in the formation of three germ layers: the ectoderm, endoderm, and mesoderm. One of the main events after gastrulation is neurulation, which is the process of the neural plate transforming into the neural tube and eventually developing into the brain and spinal cord. Following neurulation, neurogenesis commences, marking a critical period in central nervous system (CNS) development. Embryonic neurogenesis varies in its initiation and completion times across different regions of the CNS [2]. Moreover, the processes and regulatory mechanisms of neurogenesis also differ among these regions [20–22]. The cerebral cortex holds particular interest due to its crucial roles in higher-order cognitive functions in humans, such as language, perception, motor planning, and decision-making. Additionally, this region displays significant disparities between primates and rodents, underscoring the importance of studying neurogenesis specifically in primates [2,4,23]. As a result of the importance of cortical regions in the primate brain and their differences from rodent models, current research on neurogenesis primarily focuses on this region. Therefore, this section will mainly concentrate on the embryonic neurogenesis processes in cortical regions.

### Cortical neurogenesis in primates: from cell types to architecture

The neocortex originates from the pallium of the telencephalon. Once the neural tube is formed and the telencephalic primordium is established, it initially consists entirely of dividing NE cells. These proliferative cells form the ‘matrix,’ ‘germinal epithelium,’ or ‘primitive ependyma,’ which is termed the ventricular zone (VZ) by the Boulder Committee [24]. Before neurogenesis initiates, the NE cells of the VZ form a homogeneous pseudo-stratified epithelium with radial processes, dividing in a symmetric proliferative manner [25–27]. The extent of proliferation activity in NE cells is considered to have a substantial impact on brain size, as it determines the initial size of the cortical neural progenitor pool. During neurogenesis initiation, NE cells in the VZ undergo distinct morphological, molecular, and mitotic changes, gradually transforming into apical RG cells (aRG cells or ventricular radial glia cells, vRG cells) [3]. aRG cells are highly polarized, with their cell bodies localized in the VZ and two processes extending to the ventricular and pial surfaces. Their division involves interkinetic nuclear migration [28]. In the mid-to-late stages of cortical neurogenesis, aRGs begin generating basal progenitors (BPs), which progressively form the subventricular zone (SVZ) at the basal surface of the VZ where interkinetic migration is absent [29]. As only a small portion of aRGs directly produce neurons, while the rest give rise to BPs that can differentiate into neurons, BPs are considered the primary progenitor cells responsible for neuron production [30–32]. In primates, the SVZ contains two types of BPs: oRGs and basal intermediate progenitors (bIPs) [6]. oRGs were initially described as having a basal but often no apical process; however, they exhibit diverse forms in primates [4,6,33,34]. While oRGs are also found in the mouse cortex, they are exceedingly rare [35,36]. In contrast, primates have an abundance of oRGs and bIPs during development, with both displaying a high proliferative capacity [4,33]. oRG cells can divide symmetrically or asymmetrically, giving rise to a daughter oRG cell and either a bIPs or nascent neuron [4,24,37–39]. bIPs can undergo multiple divisions before generating postmitotic neurons in primate corticogenesis [33]. The SVZ in primates is expanded to accommodate the increased BP pool, and it is further divided into the inner and outer SVZ (ISVZ and OSVZ, respectively). The OSVZ is bounded by the ISVZ, and is separated by a thin layer rich in axonal fibers known as the inner fiber layer (IFL). Externally, the OSVZ is limited by an outer fiber layer (OFL) that contains the embryonic thalamic axons [4,40,41]. After approximately gestational week (GW) 17 of human development, vRG cells lose pial contacting basal processes and transform into truncated radial glia (tRG) in the VZ [27,42,43]. These tRG cells have processes that contact the ventricular surface but not the pial surface, and their processes often terminate on capillaries, particularly in the ISVZ and the inner portions of the OSVZ [27,42,43]. During this period, these tRG cells have reduced neurogenic potential, and oRG cells play a crucial role as the primary scaffold for neuronal migration into the cortical plate [38,42,44]. Similarly, tRG cells have been observed in the rhesus macaque starting from E65 [45]. However, at present tRG cells have not been reported in mice. Many questions regarding tRGs remain to be answered, such as the underlying mechanisms behind their formation, their distinct features, and the types of cells they give rise to.

To assemble the cortex architecture at the early stage, there are a diverse group of early-born ‘pioneer’ neurons (i.e. predecessor cells, Cajal-Retzius (CR) cells, and subplate (SP) cells) in the preplate, which is also known as the primordial plexiform layer [24,46–48]. In humans, these neurons occur at approximately embryonic day (E) 33 in the lateral part of the cortical wall, while in mice, it takes place at approximately E10 [49,50]. The preplate is a dynamic and largely transient compartment of heterogeneous post-migratory cells and neuropil that develops between the VZ proliferative zone and the pial surface of the dorsal telencephalon before the cortical plate (CP) appears. As cortical plate neurons (L6 layer neurons) migrate into the preplate, the preplate splits into the marginal zone (MZ) and SP zone around E13.5 in mice and the seventh to eighth week of gestation in humans [24,51,52]. The MZ is the portion of the former preplate that lies above the emerging CP and eventually develops into layer 1 of the mature cortex [24]. The MZ contains fiber bundles, CR cells, and distal dendrites of cells located beneath it. The subplate is a transient zone located below the cortical plate and above the intermediate zone in the developing cortex [24,53]. It plays a critical role in various neurodevelopmental processes involved in axon guidance and neural circuit formation [54–56]. Importantly, the subplate zone in the developing cerebrum experiences secondary expansion in humans and non-human primates [57]. The emergence of MZ and SP establishes the upper and lower borders of the cortical plate. Neurons generated in the VZ and SVZ migrate radially along the radial glia scaffold through the subplate zone and into the CP [24]. This sequential migration of distinct projection neurons forms cortical layers in an ‘inside-out’ manner [58,59]. While this basic principle is generally conserved across mammals, there are notable cellular differences in humans and non-human primates [21]. In primates, the first neurons to be generated are the deep-layer (DL) neurons, settling in layers 5 and 6 and projecting subcortically [60]. Subsequently, layer 4 neurons, responsible for receiving most of the monosynaptic connections from the thalamus, are produced. Compared to rodents, primates have an expanded and more complex thalamo-recipient cortical layer 4 [61]. Upper-layer (UL) neurons, destined for layers 2 and 3 with corticocortical projections, are then produced mainly from oRGs [6]. Upon reaching the CP, neurons receive signals to halt their migration and begin the differentiation process, involving the extension and elaboration of dendrites and the formation of synaptic connections [2]. In summary, embryonic neurogenesis during cortical development involves complex, time-dependent processes that determine the proliferation and fate of neural progenitor cells (including RGs and BPs), as well as the positioning, maturation, and functional integration of newborn neurons.

In addition, it should be noted that in the developing primate cerebral cortex, the onset of gliogenesis follows the neurogenic period, and that these processes proceed in parallel for an extended period of time [62]. However, it remains largely unclear whether and how embryonic gliogenesis affects late embryonic neurogenesis. Oligodendrocyte precursor cells (OPCs) and astrocytes are generated from RG cells after neurogenesis in a prolonged process that continues postnatally [2,63–65]. In the rhesus macaque monkey's parietal cerebrum development, cortical neurogenesis begins around E38–E40 and concludes between E70 (in the limbic cortex) and E102 (in the visual cortex) [58,66,67]; gliogenesis becomes the principal function of a robust OSVZ after E92, coinciding with gyrification initiation between E100 and E125 [68]. Additionally, oRG cells have been reported as a source of increased OPCs in the late second trimester, exhibiting molecular features similar to oRGs in humans [69]. Human OPCs can undergo several rounds of symmetric proliferative divisions, exponentially increasing the progenitor pool size [69]. These studies suggest that OSVZ gliogenesis, rather than neurogenesis, correlates with rapid enlargement and gyrification of the primate cerebrum.

### Differences in cortical neurogenesis between primates and rodents

**Figure 1.**
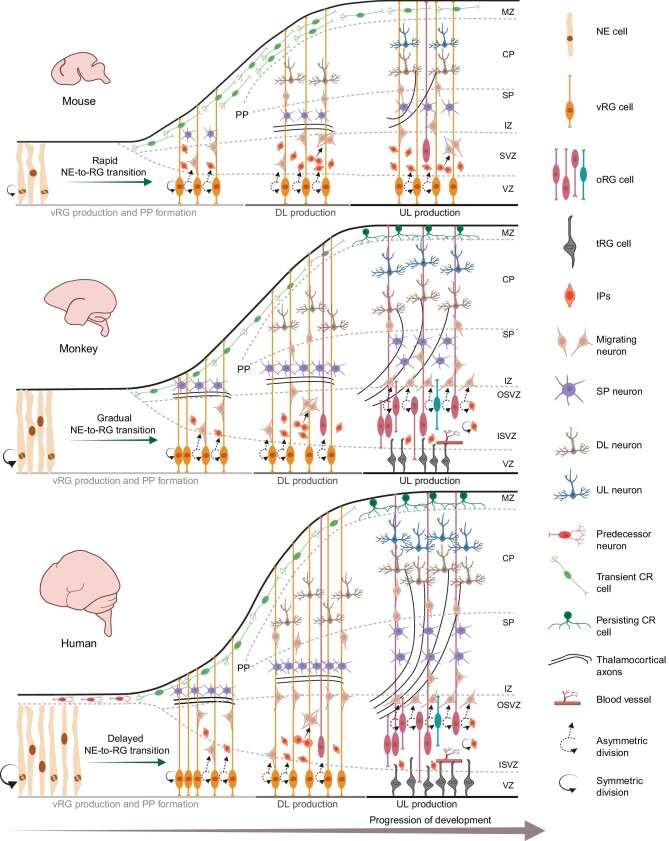
Comparison of cerebral cortical neurogenesis in mouse, monkey and human. Cortical development processes exhibit broad conservation across primates and rodents. During embryonic development, cortical layering emerges in an inside-out manner as forebrain progenitors proliferate, giving rise to distinct waves of excitatory pyramidal neurons and inhibitory interneurons. Following amplification of neuroepithelial (NE) cells and their transition into radial glial (RG) cells in the ventricular zone (VZ), RG cells undergo both symmetric proliferative divisions to expand their pool and asymmetric divisions for self-renewal and differentiation, leading to either direct neurogenesis, generating neurons, or indirect neurogenesis via intermediate progenitors (IPs), which further divide to yield pairs of neurons. However, several key differences can be highlighted in primates: predecessor neurons from subpallium in human cortex primordium prior to local neurogenesis [49]; extended NE cell expansion in monkeys and humans (proportional to arrow thickness) [3]; NE-to-RG transition: rapid in mice, gradual in monkeys, delayed in humans [3]; prominent outer subventricular zone (OSVZ) expansion in primates [6,34,231]; increased outer radial glia (oRG) cell numbers in non-human primates, especially in human cortex, driving layer 2/3 upper-layer (UL) neuron generation [60,232]; varied oRG cell types in primates [33]; an increase in the number of cell divisions for basal intermediate progenitor cells (basal IPs) and outer radial glial (oRG) cells in primates [33]; ventricular radial glia (vRG) cells shift to truncated radial glia (tRG) during the transition from deep-layer (DL) to UL neuron production in primates [42]; OSVZ axonal plexus expansion and multilaminar axonal-cellular compartment emergence in primates [106]; early thalamocortical plexus arrival in primate cortical anlage [105]; expanded subplate (SP) linked with the expansion of the axonal plexus, but not with SP cell numbers [57]; persistent Cajal-Retzius (CR) cells in primate emergence [108]. ISVZ, inner subventricular zone; IZ, intermediate zone; CP, cortical plate; MZ, marginal zone; PP, preplate. This figure was created using Biorender software.

There are significant differences in the cerebral cortex between primates and rodents. Non-human primates and humans exhibit cortical folding with sulci and gyri, whereas the brains of mice are smooth. Moreover, both non-human primates and humans possess a well-developed prefrontal cortex, including a dorsolateral prefrontal cortex, which is absent in mice [70]. The surface area of the cerebral cortex in non-human primates and humans is much larger than that of mice, with approximate ratios of 1 : 100 : 1000 (mouse:macaque monkey : human) [66]. Additionally, the cortical thickness of macaque monkeys and humans is similar, approximately twice that of mice [66]. Furthermore, the number and variety of neurons in the cerebral cortex of non-human primates and humans far exceed those found in mice [21,71,72]. Investigating the variations in cortical neurogenesis and the underlying mechanisms between species can shed light on human evolution and the development of advanced cognitive abilities not observed in mice. These species’ differences are closely related to cortical neurogenesis, which plays a crucial role in determining the number and subtypes of neurons generated. The process of cortical neurogenesis described above is largely conserved among mammals. However, there are notable disparities in cortical neurogenesis between primates and rodents, such as in the timing of neurogenesis, the proliferative capacity and diversity of neural progenitor cells, the capacity for generating diverse neuronal subtypes, and the characteristics of the neurogenesis niche (Fig. 1).

#### Longer timing of neurogenesis in primates

One significant contrast between primates and rodents lies in the timing of neurogenesis. Embryonic neurogenesis in primates extends over a longer duration compared to rodents, which is believed to contribute to the increased complexity and size of the primate cortex [2]. For example, in mice, cortical neurogenesis initiates around E8 and persists for approximately 6–7 days [21]. Conversely, in rhesus monkeys, cortical neurogenesis begins around E38–E40 and lasts for about 2 months (ending at E70 for the limbic cortex and E102 for the visual cortex) [58,66,67]. In humans, the neurogenic period in the cortex spans approximately 3–4 months [21,73].

The prolongation of cortical neurogenesis in primates is apparent across several pivotal neurogenic stages. First, the transition from NE cells to RG cells occurs at a slower pace in primates than in mice. In non-human primates and humans, this shift involves a morphological intermediate state known as transitioning NE cells [3]. Prolonged adherence of NE cells to the ventricular zone, coupled with suppressed migration and differentiation from the VZ, potentially expands the NE cell progenitor pool and contributes to a larger cortex. The elongation of this process in primates allows for an extended expansion period of NE cells. In mice, the amplification of NE cells persists for about 1 day, whereas in primates, this amplification period can span up to 2 weeks [2,21]. Second, primate cortical neural precursors undergo an extended cell-division cycle compared to rodents [21,40,74]. Within the proliferative cerebral ventricular zone of fetal rhesus monkeys, cell-cycle durations are up to five times longer than those observed in rodents [75]. Nevertheless, during the prolonged neurogenetic period in the monkey cortex, a significantly higher number of total cell division rounds occur, forming the basis for augmented cell production [75]. Third, primates exhibit an elongated postmitotic phase of fate plasticity following RG cell division, in contrast with rodents [21,76]. Postmitotic regulation of cell fates through mitochondrial dynamics is conserved in mouse and human corticogenesis [76]. However, this phenomenon occurs within a unique time frame. In human cortical progenitors, this period is further prolonged compared to mice, potentially playing a role in their increased self-renewal capabilities [76].

#### Greater diversity and enhanced proliferative capacity of neural stem and progenitor cells in primates

First, primates display a heightened capacity for NE cell expansion. The process of NE cell proliferation is believed to have a significant impact on brain size by determining the initial size of the cortical neural progenitor pool [3,77]. Notably, even before the onset of neurogenesis, the telencephalic primordium of human and macaque embryos exceeds that of mice in size [49,78]. Furthermore, the ability for NE cell proliferation in primates is largely preserved when investigating cerebral organoids *in vitro*, suggesting the presence of inherent developmental regulatory mechanisms [3].

Second, unlike rodent corticogenesis where aRGs in the VZ maintain high proliferative activity, macaque and human aRGs exhibit a decreased proliferative capacity, leading to a rapid reduction of their VZ during corticogenesis. This coincides with a significant expansion of the BPs pool in the OSVZ [4,6,34,37,41]. By GW25–GW27, while the OSVZ continues to proliferate, the human VZ reduces in size to a one-cell-thick ependymal layer [79].

Third, non-human primates and human cortical neurogenesis exhibit a more pronounced expansion of bIPs compared to rodents. While bIPs are present in significant numbers in the embryonic mouse cortex, their abundance is even higher in primates [80]. In rodents, over 95% of bIPs undergo symmetric neuronal terminal divisions [4,36,81]. In contrast, primate bIPs have the remarkable ability to self-renew for up to five successive divisions [33], indicating a parallel increase in both morphological diversity and proliferative capacity.

Fourth, notable differences between primates and rodents are observed in the characteristics of oRG cells. During mid-corticogenesis in mice, the majority of SVZ progenitors are identified as neurogenic bIPs [32,35,82,83], with oRG cells being scarcely present (<0.5%) in the mouse cortex [35,83]. In contrast, the primate OSVZ is characterized by a substantial predominance of oRG cells, constituting about 75% of all BP types [33,37]. Importantly, the macaque OSVZ showcases a diverse range of bRG morphotypes unlike rodents (Fig. 1) [33]. Through long-term *ex vivo* live imaging and unbiased sampling of cycling precursors, four distinct oRG cell morphotypes have been identified in the macaque OSVZ: basal process-bearing bRG (bRG-basal-P) cells, apical process-bearing bRG (bRG-apical-P) cells, bRG cells bearing both apical and basal processes (bRG-both-P), and transient bRG (tbRG) cells that transition between stages with or without apical or basal processes [33]. These oRG cells can undergo both symmetric proliferative divisions and asymmetric self-renewal, and they also have the ability to directly give rise to neurons [33]. They exhibit noteworthy self-renewal capabilities: *in vitro* clonal experiments have uncovered that individual oRG cells can generate hundreds of neurons [84]. The expansion of oRG cells occurs during the later stages of corticogenesis, coinciding with the generation of UL neurons [21]. Notably, these oRG cells are also capable of generating a wider variety and greater quantities of neuron types (further discussed later). Moreover, oRG cells play a crucial role in the evolution of the cerebral cortex from a lissencephalic state to a gyrencephalic state [7,8]. Importantly, the recently published single-cell atlas of early human brain development highlights the heterogeneity of human NE cells and early RGs [85,86]. These distinct characteristics within neural stem and progenitor cells in primates significantly contribute to the expanded cortical surface area and thickness observed in them.

#### Enhanced neuronal diversity and output in primates

The third significant difference in cortical neurogenesis between primates and rodents is reflected in the type and number of neurons produced by neural stem and progenitor cells. The neocortex is comprised of six layers, housing both long-distance-projecting, excitatory pyramidal neurons and locally projecting, inhibitory interneurons. Recently, through a combination of approaches including single-cell transcriptomics, spatial transcriptomic analysis, electrophysiological studies, and morphological profiling, researchers have unveiled significant cellular distinctions in primates compared to rodents [87–90]. There is enhanced heterogeneity and diversity among UL and DL neurons in the primate cortex [87–90]. In humans, researchers have identified at least five distinct subtypes of UL neurons, whereas mice have only three [87,91]. Notably, Betz neurons, a subtype of DL corticospinal neurons responsible for fine motor control, are enriched in primates [88]. Comparing transcriptomic data across human, macaque, and mouse cortices unveiled primate-specific L4/5 IT glutamatergic neurons enriched in cortical layer 4, with their marker genes expressed in a region-dependent manner [90]. Previous viewpoints have posited that excitatory neurons originate from progenitor cells in the developing pallium, while γ-aminobutyric acid–expressing (GABAergic) interneurons derive from the ganglionic eminences [37,92]. However, recent research has reshaped this notion, revealing that GABAergic neurons in the primate brain cortex may arise from cortical precursors within OSVZ. Cortical precursors for GABAergic neurons have been identified in the developing brains of humans and macaques [93–96]. Notably, these precursors are capable of generating calretinin neurons, which represent the predominant category of interneurons in primates (12%) [97]. *In vitro* experiments have further shown that human cortical progenitors, unlike those of mice, exhibit the capacity to generate GABAergic neurons with transcriptional features and morphologies similar to cortical interneurons [98,99]. Recent studies also illuminated that the diversity of cortical neurons is established early during the precursor stages of pallium and ganglionic eminence formation [92]. Consequently, the increased neural diversity observed in primates could be attributed to the heightened diversity and differentiation capacity of neural stem and progenitor cells found in primates, enabling differentiation into a broad spectrum of cell types. These findings provide fundamental insights into brain development and evolution.

#### Distinctive traits of the neurogenic microenvironment in primates

The dynamic activity exhibited by the processes of aRG cells and oRG cells facilitates the sampling of the cerebral cortical microenvironment, which extends from the pia to the ventricular surface. Through this intricate mechanism, signals from both pre- and postmitotic cells, along with those present in the cerebrospinal fluid, are integrated [100,101]. Consequently, microenvironmental changes play a significant role in neurogenesis, influencing neural stem cell proliferation and differentiation. Research has highlighted notable disparities in the cortical neurogenic microenvironments between rodents and primates, potentially contributing to distinct neurogenic outcomes among species.

In primates, the OSVZ displays several distinctive characteristics. First, it offers a microenvironment enriched with extracellular matrix (ECM), in contrast to the mouse SVZ, where ECM components are downregulated compared to the VZ [102]. Second, the trajectory of embryonic thalamic axons in the developing cortex varies significantly between primates and rodents. Prominent axon tracts from dorsal thalamic nuclei innervate the developing cortex. These axons release extrinsic factors, such as basic fibroblast growth factor (bFGF) and VGF nerve growth factor inducible, which foster the proliferation of cortical precursors [103,104]. The thalamic axons’ arrival in the developing cortex of primates precedes that in rodents, occurring even before stage CS18 of human development [105]. By this stage, fibers from the primitive internal capsule have reached and likely penetrated the developing cerebral vesicle [105]. Additionally, in primates, thalamic axons persistently remain in proximity to the OSVZ and OFL [41]. Notably, there is an expansion of the OSVZ axonal plexus and the emergence of a multilaminar axonal-cellular compartment in the fetal human cerebrum [106]. Conversely, in fetal mice, thalamocortical axons are positioned more dorsally, distant from SVZ [41,103].

The processes of vRGs and oRGs can extend into the SP and MZ regions, facilitating information exchange that influences the neurogenic process. However, these regions also exhibit significant differences between rodents and primates. Apart from the distinct characteristics of CR cells in the MZ (as described above), the subpial granular layer (SGL) is found in humans and monkeys [107]. In contrast, rodents lack a clearly identifiable SGL [108]. While still requiring further investigation, this structure is believed to serve as an additional source of CR cells and interneurons in primates [109]. Similarly, distinct characteristics are evident in the primate SP beyond those already described for SP neurons [54]. In rodents, the SP is relatively small and exhibits uniform width. The murine SP compartment undergoes a minimal size increase during embryonic development and is not always clearly distinguishable in histological sections [54]. In contrast, the subplate in primates is notably thicker and varies in thickness based on the brain region [57]. This increased subplate thickness in primates primarily results from a substantial influx of fibers, leading to the dispersion of early-generated subplate cells after completing active migration [57].

#### Mechanisms underlying primate-rodent differences in cortical neurogenesis

In the intricate landscape of neurodevelopment, the regulatory mechanisms shaping the divergent pathways of cortical neurogenesis in primates and rodents stand as a captivating enigma awaiting exploration. Delving into this realm of inquiry promises not only to unravel the profound disparities that have shaped the evolution of their brains but also to offer profound insights into the fundamental forces driving the complexity of neural development across species.

##### Molecular mechanism.

Primates exhibit species-specific differences in embryonic neurogenesis due to the molecular regulation mechanisms. These differences contribute to the diversity of brain structure and function among primates, as evidenced in reviews and articles [60,65,70,110]. Many of the key genetic regulators of cortical development are conserved throughout the vertebrate lineage [111]. However, their patterns of expression exhibit significant variations across species and even within distinct brain regions of the same species. These differences in gene expression regulation are recognized as significant drivers of phenotypic diversity during evolution. For instance, the zinc-finger transcription factor ZEB2 has been identified as a driver of the NE-to-RG cell transition, and its delayed onset in humans contributes to human-specific neocortical expansion [3]. The expression of the proneural transcription factor *BRN2* occurs earlier in telencephalic development in monkeys than in mice, as *BRN2* is strongly expressed in the early aRG cells of the VZ at E36 in monkeys, while it marks late RGs and upper-layer cortical neurons in mice. In contrast to its limited effects on mouse brain development, a *BRN2* biallelic knockout in cynomolgus monkeys is lethal before midgestation [112]. Research has uncovered various factors contributing to differential gene expression regulation, including primate-specific miRNAs, human accelerated regions (HARs), human gained enhancers (HGEs), 3D nuclear organization of DNA, and chromatin structures. These elements collectively contribute to the disparities observed in cortical neurogenesis between primates and rodents (as reviewed in [4,21]). Of particular interest, recent studies have revealed that endogenous retroviruses (ERVs) can also influence neural development processes. Studies have revealed region-specific expression of ERVs in the developing human brain, forming transcriptional networks via interactions with proteins like TRIM28 [113]. ERVs also impact neural differentiation through TUT7 and NAT1 proteins [114], while the envelope protein of HERV-K (HML-2) maintains stemness and triggers specific signaling pathways crucial for neurodevelopment [115]. These studies collectively underscore the central importance of ERVs in sculpting neural differentiation and development. ERVs play a role in forming gene regulatory networks, affecting RNA processing, and sustaining stemness. However, the specific impact of ERVs on the process of cortical neurogenesis and their underlying mechanisms require further exploration.

The increase in gene copy number and variations in protein function resulting from mutations in existing genes in primates contribute significantly to the disparities in cortical neurogenesis between primates and rodents. Frequent genomic duplications in primate and human lineages, exemplified by DUF1220, have introduced pivotal changes to the cortical neurogenesis program [116,117]. Amino acid substitutions further modify protein function, evident in instances like FOXP2, a transcription factor heavily expressed in the developing and adult human neocortex, which is associated with language and vocal learning [118]. The amino acid substitutions in its DNA-binding motif influence neurogenesis and synaptic plasticity [119,120]. Another illustrative case is transketolase-like 1 (TKTL1), possessing a lysine-to-arginine substitution, augments the abundance of basal radial glia in modern humans, which is pivotal for enhanced neuron generation [121].

The driving force behind differences between primate and rodent neurogenesis can also stem from novel genes, often arising due to extensive segmental genomic duplications or retrotransposition events within primates [122]. A substantial number of genes unique to humans, hominoids, or other primates appear to have played a pivotal role in the evolutionary expansion of the cerebral cortex. Notable examples include ARHGAP_11_B (Rho GTPase-activating protein 11B), *NOTCH2NL* (Notch 2 N-Terminal Like), *CROCCP2* (ciliary rootlet coiled coil pseudogene 2), *TBC1D3* (TBC1 domain family member 3), and TMEM14B (transmembrane protein 14B), all of which contribute to the enlargement of basal progenitor populations, including oRG cells [8,123–126]. Among these, inactive *ARHGAP_11_B* emerges as a human-specific gene resulting from a partial duplication of *ARHGAP_11_A*. It contributes to the augmentation of the human neocortex by fostering the generation of bRG cells while suppressing neural progenitor cell differentiation [127,128]. The hominoid-specific gene *TBC1D3* stimulates the generation of basal neural progenitors and triggers cortical folding in mice [129]. In the primate context, TMEM14B, expressed within oRG cells of the human neocortex, induces IPs and oRG expansion by promoting G1/S transitions and gyrification when introduced into mice [8].

##### Cellular mechanism.

Cellular processes are also associated with the species-specific features of cortical neurogenesis. Mitochondrial dynamics in postmitotic cells regulate neurogenesis and show differences between humans and mice [76]. Augmenting mitochondrial metabolism has demonstrated its potential to expedite human neuron maturation both *in vitro* and *in vivo*, leading to advanced development; conversely, suppression of this process in mouse neurons results in reduced maturation rates [130]. Remarkably, human cells exhibit an approximately twofold greater protein stability and an extended cell cycle duration compared to mouse cells [131]. Lysosome-mediated protein degradation also displays notable variation across mammalian species [132]. Moreover, diminished rates of protein translation can directly influence the timing of cortical neurogenesis [21,133,134]. Mitotic spindle orientation and cell adhesion are also reported to regulate cortical neurogenesis and show species-specific features (reviewed in [40,135]). Cumulatively, these findings underscore the likelihood that variations in cellular processes, encompassing mitochondrial metabolism, protein turnover, cell division and cell adhesion, significantly shape species-specific temporal developmental disparities.

### Non-human primates hold great promise for understanding human neurodevelopmental diseases

The development of the brain involves a sequential series of events, including neurogenesis, new neuron migration, differentiation/maturation, and the establishment of synaptic connections [136]. Neurogenesis generates undifferentiated neural stem cells that serve as the foundation for brain development. Through proliferation, these stem cells give rise to progenitor cells, which subsequently differentiate into specialized neurons. The timing and location of neurogenesis are pivotal in determining neuronal migration paths. Guided by molecular cues and structural scaffolding, newly formed neurons migrate from their birthplace to precise destinations, ensuring organized positioning within the developing brain. In these designated locations, neurons undergo further differentiation and maturation, acquiring distinct functional and morphological characteristics. The establishment of synaptic connections enables neuronal communication and the formation of functional circuits. Neurogenesis sets the stage for subsequent processes, providing neuronal diversity and a population crucial for migration, differentiation, and the establishment of intricate synaptic connections, ultimately shaping the complex architecture and functionality of the mature brain. Malfunctions in any of these steps can lead to neurodevelopmental disorders, such as autism spectrum disorders, schizophrenia, intellectual disability, microcephaly, hemimegalencephaly, and lissencephaly.

Most of our understanding of the molecular mechanisms underlying human neurodevelopmental diseases is from rodents. However, differences in the regulation of gene expression, neurodevelopmental processes, and brain structures between rodents and humans make it difficult to model human neurodevelopmental diseases in rodents. As a result, many studies using rodent models have not been successfully translated into clinical applications, and drug development based on these models has faced significant challenges. Recent advancements in human brain organoid culture have provided an efficient means to model structural malformations that include microcephaly, lissencephaly, and macrocephaly. However, brain organoids have limitations in modeling disorders that affect neural networks (reviewed in [137]), and their developmental capacity needs to be improved. Non-human primates, with their close similarity in gene expression patterns and brain architecture to humans, offer a valuable avenue for studying human neurodevelopment and diseases. Compared to other animal models, they show great promise in bridging the gap between basic research and clinical translation. The prolonged stages of cortical cell production observed in non-human primates provide a unique opportunity for detailed temporal investigations into brain structural development, molecular regulatory mechanisms, and the impact of critical windows during CNS development in response to external stimuli.

Recent progress in generating genetic mutants in non-human primates has created new opportunities to investigate neurodevelopmental disorders in humans by using genetically modified non-human primates as models. For example, Rett syndrome (RTT) is a neurodevelopmental disorder caused by a mutation in the *MECP2* (methyl-CpG-binding protein 2) gene. Although mouse models have been used to study the disorder, they do not fully represent the complete range of symptoms observed in human patients, and male mice with the mutation are viable [138]. In contrast, a *MECP2* mutant cynomolgus monkey model has been developed, with females showing similar symptoms to human RTT patients, including reduced social interaction, stereotyped repetitive behaviors, and decreased sensitivity to sensory stimuli [139]. Mutant monkeys also exhibited structural MRI (magnetic resonance imaging) abnormalities, reduced heart rate, and changes in immune and RNA processing pathways. This model demonstrates the potential value of genetically modified non-human primates for studying RTT, with future research expected to further elucidate disease mechanisms and uncover potential therapies. Mouse models have limitations in simulating aspects of autism spectrum disorder (ASD), while monkeys can mimic human ASD symptoms and have helped researchers to discover disease mechanisms. Mutations in the *SHANK3* (SH3 and multiple ankyrin repeat domains protein 3) gene cause ASD and Phelan-McDermid syndrome, and mouse models have provided insights into *SHANK3* function. However, heterozygous *SHANK3* knockout mice only show mild or no phenotypes [140,141]. To address this, *SHANK3* mutant cynomolgus monkeys were generated using CRISPR/Cas9, and both full knockout and heterozygous mutant *SHANK3* monkeys exhibited multiple behavioral abnormalities, altered global and local functional connectivity, and potential biomarkers consistent with human ASD patients [142]. *CHD8* deficiency-mediated ASD symptoms and macrocephaly were also investigated in monkeys, revealing that gliogenesis is crucial for brain size in primates and that abnormal gliogenesis may contribute to ASD [143]. These monkey models can facilitate the discovery of biomarkers and preclinical evaluation of therapeutics for ASD. *MCPH1* (microcephalin 1), also known as BRIT1 (BRCT-repeat inhibitor of hTERT expression 1), plays a crucial role in the enlargement of the primate brain, and its mutation leads to microcephaly accompanied by mental retardation. *MCPH1* deficiency in mice led to small skull sizes, hearing impairment, or smaller brains [144,145]. Furthermore, in the mouse models not all *MCPH1* mutants replicate the severe human brain size reductions, and neurological symptoms are absent [146,147]. Importantly, the microcephaly phenotype of *MCPH1mt/mt* monkeys closely resembles that caused by mutant human *MCPH1* [148].

Although some non-human primate models of neurodevelopmental disorders have been successfully constructed and can simulate human diseases well, there are still many human neurodevelopmental diseases with a lack of primate models, and the mouse models of these diseases differ greatly from human diseases. For example, lissencephaly is a rare genetic disorder that affects brain development, resulting in the absence or underdevelopment of folds in the brain's surface. Mutations in the *LIS1* (lissencephaly-1) or *DCX* (doublecortin) genes are known to cause lissencephaly [149]. *LIS1* is involved in the regulation of microtubules that are essential for neuronal migration during brain development, while *DCX* codes for a protein that plays a crucial role in neuronal migration. Although mouse models with mutations in these genes have been created, they do not fully recapitulate the human disease phenotype. Specifically, lissencephaly in humans is characterized by a smooth brain surface, whereas the surface of the mouse brain is naturally relatively smooth. It also lacks prominent gyri and sulci. While mutations in *LIS1* and *DCX* are associated with the disorder, the exact mechanisms by which they cause lissencephaly are not fully understood. Therefore, developing non-human primate models of lissencephaly might help explore the mechanisms of the disease in more depth in the future. These gene mutation related neural developmental diseases and their phenotypes in human, monkey, and mouse models are summarized in Table 1.

**Table 1.** Gene mutation-related neural developmental diseases can exhibit diverse phenotypes in mouse, monkey, and human models.

Mutant gene	Neurodevelopmental disorder	Mouse models	Monkey models	Human phenotypes
*MECP2*	Rett syndrome (RTT)	• Both males and females are viable • Neurological symptoms typically appear at 5–6 weeks • Exhibiting a distinct phenotype compared to the human disease [138]	• Males exhibit embryonic lethality • Females show physiological, behavioral, and structural abnormalities • Resembling the clinical manifestations of RTT patients [139]	• Patients are females with heterozygous mutation • Loss of voluntary movements between 6 and 18 months • Exhibiting mental retardation [139]
*SHANK3*	Autism spectrum disorder (ASD) and schizophrenia	• Synaptic transmission defects (InsG3680 mutation) • Impaired juvenile social interaction • Heterozygous mutants only show mild phenotypes (R1117X mutation) [140,141]	• Full knockout and heterozygous monkeys exhibit multiple behavioral abnormalities • Altered global and local functional connectivity, resembling human ASD patients [142]	• Aberrant synaptic connections • Experiencing defective development of neural networks • Abnormal neural synchronization [234,235]
*CHD8*	ASD symptoms and macrocephaly	• Abnormal behavior and enhanced neuronal activation in males • Reduced baseline neuronal activity in female [236]	•Enhanced gliogenesis (astrocytes and oligodendrocytes) • Increased white matter volume [143]	• Autism • Language disability and sleep disorder • Macrocephaly [237,238]
*MCPH1*	Microcephaly	• No neurological symptoms • Not all mutants show reduced brain size [144,146,147]	• Closely resembling *MCPH1* mutant microcephaly in humans [148]	• A smaller brain size • Reduced neural progenitor proliferation [148]
*LIS1*	Lissencephaly	• Homozygous-null mice exhibit early embryonic lethality • Neuronal migration defects [149,239]	• Lack of models	• Lissencephaly • Abnormal progenitor proliferation and neuron migration • Delayed brain development [240]
*DCX*	Lissencephaly	• KO shows abnormalities in hippocampal CA3 pyramidal cell lamination • Suffering from spontaneous epilepsy [241]	• Lack of models	• Lissencephaly • Subcortical band heterotopia • Neuronal migration defects [242]

## ADULT NEUROGENESIS IN PRIMATES VERSUS RODENTS

Adult neurogenesis is the process of generating new neurons in the adult brain, which is widely conserved across different species, including fish, birds, and mammals [150,151]. As evolutionary status increases, the regions of adult neurogenesis become more restricted, and the capacity gradually decreases [152,153]. In mammals, adult neurogenesis was first discovered in the 1960s by Joseph Altman [154,155]. However, its existence was not confirmed until the 1990s using BrdU labeling and immunostaining with neuronal maker [156–158].

### Evident adult neurogenesis in rodents

In recent decades, the existence of adult neurogenesis in rodents has been widely acknowledged and extensively studied. Numerous research papers and reviews have documented the characteristics and regulatory mechanisms associated with this phenomenon [19,152,159,160]. In this context, we will only offer a concise overview. In the adult rodent brain, there are limited numbers of neural stem cells located in two specific niches that support neurogenesis: the SVZ in the lateral ventricles and the SGZ in the hippocampus [159]. The SVZ produces new interneurons for the olfactory bulb, contributing to odor discrimination; the SGZ generates new granule cells in the dentate gyrus of the hippocampus, which is essential for learning and memory [19,161]. Recent studies have also suggested the presence of adult stem cell populations and potential neurogenesis in other brain regions, such as the hypothalamus, striatum, substantia nigra, cortex, and amygdala under stroke/ischemia and neurodegenerative disorders [162,163]. While the level of neurogenesis decreases with age in the rodent brain [164], considerable amounts of proliferating NSCs and newborn neurons are sustained in adulthood [165,166].

### Adult neurogenesis in non-human primates persists throughout their lifespan

In contrast to rodents, research on adult neurogenesis in primates has been limited due to technological, material, and ethical constraints. Compared to human studies, it is more feasible to conduct cell labeling and marker immunostaining experiments in non-human primates to track adult neurogenesis. Using the BrdU labeling method, newborn neurons were discovered in the hippocampus of both marmoset monkeys and Old-World monkeys [167,168]. The rostral migratory stream (RMS), a migratory route for neuronal precursors from the subventricular zone to the olfactory bulb, has also been observed in the adult monkey brain [169]. Moreover, the presence of newborn neurons in the amygdala, neocortex, piriform cortex, and adjoining inferior temporal cortex of adult primates has been reported, although some findings remain controversial [170–172]. Compared to other brain regions, a significant number of reports regarding adult hippocampal neurogenesis emerged after the 1990s, which have been well reviewed [173,174]. Studies have shown the occurrence of adult hippocampal neurogenesis in some non-human primate species (i.e treeshrews, marmosets, and macaque monkeys) using various techniques, such as BrdU labeling and marker immunostainings for proliferating cells (e.g. proliferating cell nuclear antigen, PCNA), immature neurons (e.g. class III β-tubulin, TuJ1, and Turned on after division 64 kDa protein, TOAD-64) and mature neurons (neuron-specific enolase, NeuN) [167,168,175]. Moreover, several reports have indicated that adult hippocampal neurogenesis in non-human primates can be affected by stress [176], antidepressants [177], and ischemia [178–180]. Recent studies utilizing single-cell sequencing techniques have confirmed the existence of neural stem cells and newborn neurons in the adult monkey hippocampus [11,17,181,182]. These studies have also shown that neural stem cells isolated from the adult monkey hippocampus can be cultured *in vitro* while retaining their self-renewal and differentiation capabilities into neurons [181]. However, the rate of adult neurogenesis in monkeys is much lower than in mice, with a noticeable decline during infancy and early childhood [14]. In contrast, the maturation of neurons is relatively prolonged, taking six months or more [183]. The precise functions of these newborn neurons, including their potential involvement in memory and learning [184], remain to be further investigated, particularly in comparison to their rodent counterparts [185]. While adult neurogenesis in non-human primates persists throughout their lifespan at a low level, adult neurogenesis in humans has been a topic of controversy for decades. We will delve into this topic in the subsequent discussion.

### Adult neurogenesis in humans needs more scrutiny

As mentioned above, various sources of data, including immunostaining, single-cell sequencing, and stem cell isolation and cultivation, have substantiated the existence of adult neural stem cells and neurogenesis in monkeys. Nevertheless, the occurrence of neural stem cells and neurogenesis in the adult human brain has been debated. In humans, the turnover of olfactory neurons is negligible after infancy [186,187]. Instead, activated NSC and neuroblast cells can be found in the subventricular zone and the neuroblast cells migrate toward the nearby striatum [188,189]. The existence of adult hippocampal neurogenesis in humans is more controversial. Some research laboratories have reported that hippocampal neurogenesis terminates in humans after childhood [10,14,190], while others have observed its persistence into old age and even its detection in individuals with neurological disorders [9,13,15,16]. These studies mostly rely on tissue immunostaining and quantification. Because the accuracy and reliability of tissue immunostaining results can be influenced by various factors, including the sensitivity and specificity of the antibodies used, the postmortem interval of tissue samples, the methods of fixation, and the staining protocols, discrepancies have been observed among these studies.

The emergence of single-cell transcriptomic analysis has been expected to provide new opportunities to resolve the aforementioned discrepancies. By using single-cell transcriptomic sequencing, cell populations and markers can be identified to construct the developmental trajectories. However, the controversy with adult human hippocampal neurogenesis persists despite the use of single-cell sequencing techniques. While Franjic *et al.* did not find neurogenic lineages in adult human hippocampal tissues [11], several research groups, including ours, have identified neural stem cells and immature neurons in the adult human hippocampus using single-nucleus transcriptomic sequencing [17,18,191]. By analyzing single-cell sequencing data obtained from human hippocampal samples of varying ages, these three studies have demonstrated that neural stem cells and neurogenesis do exist in the adult human hippocampus, and also identified STMN1 (stathmin 1) and STMN2 (stathmin 2) as immature neuron markers in humans. Hongjun Song's group further validated the neurogenic capacity of the adult human hippocampus by confirming the existence of proliferating neural progenitors and newborn granule neurons in cultured surgical specimens. Notably, Song's group found a significantly higher population (3%–8%) of immature granule cells in humans compared to adult monkeys and mice [14,18,192], but with very few progenitors (0.005%–0.015%). This observation could potentially suggest that neurons in humans have a longer maturation period compared to non-human primates and rodents [183]. However, several reports did not find comparable amounts of immature granule cells based on *DCX* or PSA-NCAM (polysialylated form of neural cell adhesion molecule) staining in middle aged and old humans [9,16,191]. Also discussed by Tosoni *et al.* [193], factors such as sample processing, experimental design, computational analysis, inter-individual variability, and co-existing pathologies may directly interfere with the neurogenic process. Together, given the ongoing controversy and unresolved challenges, adult human neurogenesis requires more scrutiny, and we must consider the functional significance of such a small population of cells.

### Characterizing neurogenic lineages in adult primates requires identifying specific markers

**Figure 2.**
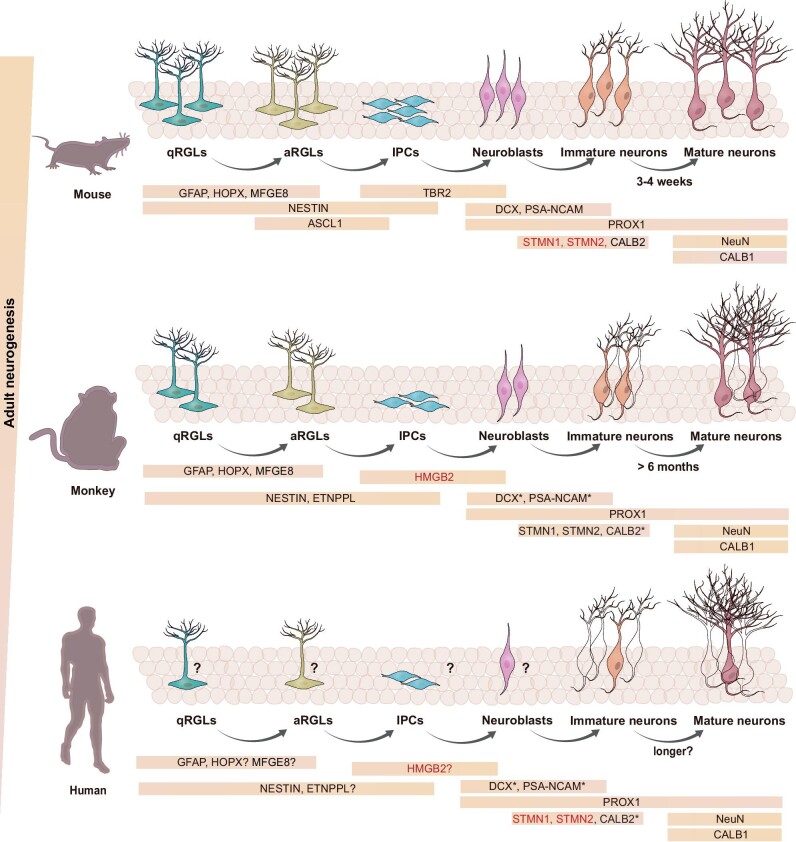
Comparison of hippocampal neurogenesis capacity and markers in mouse, monkey, and human. Neurogenic lineages and their corresponding markers in the adult hippocampus are presented based on previous studies [18,19,159,195–197,233]. The traditional markers that were first discovered in mice are shown in black, while the markers that were newly found in primates are shown in red, indicating the corresponding neurogenic populations. The asterisk symbols (*) indicate the markers that are not specific to newborn neurons in primates. The question marks (?) indicate markers that have been reported in monkeys but need further validation in humans. The colorless neurons represent the presence of long-lasting immature neurons and their potential to mature in primates. This figure was created using Adobe Illustrator.

During the process of adult neurogenesis, neural stem cells undergo proliferation, differentiation, and maturation stages, ultimately transforming into functional neurons. Each stage is characterized by the expression of distinct marker proteins in different cell types, providing indicators of the adult neurogenesis process. In extensively studied mouse hippocampus, quiescent neural stem cells express GFAP, HOPX (homeodomain only protein), and Nestin (intermediate filament protein), while activated neural stem cells begin to express BLBP (brain lipid-binding protein), Ascl1(achaete-scute homolog 1), EGFR (epidermal growth factor receptor), PCNA, and Ki67. Neural progenitor cells highly express Tbr2, and as they differentiate into neuroblasts and become dentate granule neurons, they express Prox1 (Prospero homeobox 1), *DCX*, PSA-NCAM and CALB2 (calretinin). Finally, during the maturation process, NeuN and CALB1 (calbindin 1) is upregulated [19,159,194–197] (Fig. 2). However, in the context of adult neurogenesis in primates, do cells at different stages express similar markers as rodents or display species-specific differences? Currently, research on adult neurogenesis in primates is still limited due to the lack of reliably identified markers. Several studies including our own indicated that some markers commonly used in mice may be non-specific in primates. For instance, *DCX*, PSA-NCAM, and CALB2 are widely used to identify newborn neurons in mice, but they have also been found to be expressed in non-neurogenic brain regions and mature interneurons in primates [11,198–202]. We have also shown that *DCX* and CALB2 are highly expressed in mature interneurons in humans [191]. As most current research still relies on insights gained from mouse models, there is a pressing need to develop markers that specifically indicate neural stem cells and newborn neurons in primates.

Recent advancements in single-cell sequencing analyses have provided valuable insights into identifying markers for neural lineages in primates. For example, ETNPPL (ethanolamine-phosphate phospho-lyase) is identified as a primate-specific marker for neural stem cells [17], HMGB2 (high mobility group protein B2) for IPs [181], and STMN1 and STMN2 as markers for newborn granule neurons [17,18] (Fig. 2). These findings highlight the potential of single-cell sequencing analysis to unveil new marker genes that can be used in primate studies, enhancing our understanding of adult neurogenesis in these animals. Nevertheless, it is crucial to carefully evaluate the specificity of existing markers in characterizing the process of primate adult neurogenesis. Additionally, further verification of the reliability of newly identified markers is necessary. By combining the markers identified from transcriptomics, proteomics, epigenomics, and metabolomics, we can better explore the similarities and differences between adult neurogenesis in primates and rodents with greater accuracy.

### Monkey models for understanding adult neurogenesis and its related diseases

As discussed above, the scarcity of human samples and limited interventions have been persistent obstacles in definitively resolving the controversy surrounding adult neurogenesis. With this predicament, non-human primates, especially monkeys, provide a bridge for understanding adult neurogenesis in the human brain due to their high similarity to humans and easier access to materials. Studies on monkeys have reliably shown that adult neurogenesis in primates declines significantly after birth [14,17], and that immature neurons in primates undergo a longer maturation period [183]. Moreover, markers that characterize neurogenesis in rodents may not be specific in primates [11,198–202]. The striking similarity between monkeys and humans enables us to investigate the underlying mechanisms of neurogenesis in primates and explore its potential therapeutic application in the treatment of human neurological disorders. For example, we could identify reliable primate-specific markers of neurogenic lineage and investigate the functions of primate adult neurogenesis by using monkey models. By manipulating key regulatory factors in the monkey models, we can then investigate whether these factors can promote adult neurogenesis in primates. These studies can provide a solid foundation for human neurogenesis research. However, it is important to note that research on monkeys cannot fully address the challenges faced in human studies, as adult neurogenesis in non-human primates is higher than that in humans [11,14,17,18,181,182]. The question remains whether the low levels of neurogenesis observed in humans hold significance or only play a role in specific circumstances [203,204] such as injury, neuropsychiatric disorders, or environmental stimuli, which necessitates further investigation.

It is known that the neurogenesis capacity and neurogenic lineage marker proteins of the adult brain under normal physiological conditions show some consistency as well as differences between rodents and primates. However, what happens to adult neurogenesis under pathological or disease-related conditions? Can adult neurogenesis regulate brain function in these states? These questions are of great interest and importance in the field. In conditions such as epilepsy and stroke, mouse models have shown a significant increase in neurogenesis [205,206]. Similarly, patient samples have indicated that stroke and epilepsy can induce neurogenesis [205,207], showing a high degree of consistency between mouse models and human patients. Nevertheless, the changes in adult neurogenesis are variable in different neurodegenerative diseases between mouse models and human patients. In mouse models of Alzheimer's disease (AD) with overexpression of either amyloid precursor protein (APP) or presenilin, inconsistent changes in neurogenesis have been observed [208]. In mouse models of Parkinson's disease (PD) that overexpress wild-type α-synuclein, the survival of newborn neurons is significantly reduced, and the expression of an α-synuclein mutant inhibits cell proliferation [209]. R6 transgenic mice exhibit several symptoms and signs of Huntington's disease (HD) the same as in humans [210], including neurological and endocrine changes. The reduction in neurogenesis of R6 mice is evident in the dentate gyrus, but the number of newborn neurons is normal in the subventricular zone and olfactory bulb [211]. Unlike mouse models, patient samples have shown that neurogenesis is decreased in individuals with AD but increased in those with PD, amyotrophic lateral sclerosis (ALS), and HD [9,13,15,16]. Therefore, using mouse models to study the relationship between adult neurogenesis and neurodegenerative diseases may not accurately represent the situation in the human brain and could potentially lead to misunderstandings about the underlying mechanisms.

Furthermore, compared to the scarcity of human tissue samples, the use of non-human primates allows for gene editing and the construction of models that can more closely simulate human neurological diseases. This provides valuable opportunities to advance our understanding and develop innovative therapeutic approaches for neurological diseases. Non-human primate models, obtained through natural occurrences, genetic manipulation, and special treatments, have been flourishing in recent years. These models can mimic age-related cognitive decline, neurodegeneration (AD, PD, and HD), epilepsy, stroke, autism, and psychiatric disorders [212–214]. For instance, non-human primate models of AD have been established by intracerebral delivery of amyloid-β oligomers [215–217] or through PSEN1 mutation by TALEN [218]. To mimic PD, chronic low-dose MPTP (1-methyl-4-phenyl-1,2,3,6-tetrahydropyridine) or MPP+ (1-methyl-4-phenylpyridinium ion, a toxic metabolite of MPTP) has been employed in monkeys, leading to cognitive impairments such as deficits in working memory, cognitive flexibility, and visuospatial attention [219–223]. By expressing exon 1 of the human gene encoding huntingtin with 29 CAG repeats regulated by the human polyubiquitin C promoter, a transgenic HD monkey model has been developed. It shows similar disease patterns to HD patients, including a decrease of N-acetylaspartate (NAA), neuronal damage/loss in the striatum, and progressive cognitive and motor impairment [224,225]. In addition to the above discussion, various non-human primate models with brain disorders have been well summarized by Scott and Bourne [214]. These non-human primate models are summarized in Table 2 and they can provide ample opportunities for observing and verifying the relationship between neurogenesis and neurological disorders in a more controllable environment.

**Table 2.** Changes in adult neurogenesis in neurodegenerative disease models and clinical samples.

Neurodegenerative disease		Alzheimer's disease (AD)			Parkinson's disease (PD)		Huntington's disease (HD)
	Models	APP single-transgenic [208]	PS1 (presenilin-1) single-transgenic [208]	APP/PS1 double-transgenic [208]	Overexpression of α-synuclein WT [209]	Overexpression of α-synuclein A53T [209]	R6 transgenic mice [210,211]
Mouse	Adult neurogenesis-related phenotypes	Newborn neuron survival↓	No effect	Newborn neuron survival↓	Newborn neuron survival↓	Cell proliferation↓ Newborn neuron survival↓	Neuroblasts ↓ Newborn neurons↓
	Models	AβO injection into lateral ventricle [215,216]	AβO injection into cerebral parenchyma [217]	PSEN1 exon 9 deletion [218]	Intravenous injection of MPTP [219,220,222,223]	Injection of MPP+ into the putamen [221]	Expression of human HTT exon 1 with 29 CAG repeats [224,225]
Monkey	Adult neurogenesis-related phenotypes	Synapse ↓ Microglia↑ Astrocytes↑	Microglia↑ Astrocytes↑ Specific neurons↓	Under investigation?	Attention ↓ Working memory↓	Dopamine ↓ Motor functions↓	Neuronal damage↓ Cognition ↓ Motor functions ↓
	Clinical samples	AD patients [9,13,15,16]			PD patients [15]		HD patients [15]
Human	Adult neurogenesis-related phenotypes		Cognition ↓ Immature neurons↓ Mature neurons↓		Cell proliferation ↑ Immature neurons↑		RGLs↑ Immature neurons↑

## LIMITATIONS OF NON-HUMAN PRIMATE MODELS

Since non-human primates are phylogenetically closer to humans than rodents, they are expected to answer the above-mentioned questions, provide valuable insights for understanding human neurogenesis, and further clarify the mechanisms that control the specificity of neurogenesis in primates under both physiological and pathological conditions. Nevertheless, non-human primate models have limitations in investigating human-specific features and mechanisms. These include human-specific gene regulatory elements, protein coding mutations, novel genes, and gene expression patterns [21]. The specific roles of these various examples cannot be fully replicated in non-human primates. For instance, HARs are *cis*-regulatory elements that have undergone rapid evolutionary changes specifically in the human genome compared to other species [226,227]. Human HAR5 regulates the expression of *FZD8* (frizzled class receptor 8), leading to increased RG cell proliferation and cortical size [228]. Regarding adult neurogenesis, monkeys also cannot fully address the challenges faced in human studies, as the level of adult neurogenesis in non-human primates remains higher in most reports [11,14,17,18,181,182]. As discussed previously and summarized here, these human-specific features imply that research in non-human primates cannot completely replace research in humans. Therefore, it is important to learn the similarities and differences in embryonic and adult neurogenesis among rodents, monkeys, and humans to help us choose the appropriate animal models to answer specific research questions.

Importantly, when we use non-human primate models to answer the scientific questions, we should take responsibility and consider animal ethics and welfare, which have been well discussed [229,230]. Prior to initiating projects, it is crucial to provide a comprehensive and careful evaluation for the necessity and suitability of using non-human primate models. This includes having a scientific understanding and justification for why a non-human primate model is the most suitable tool. Additionally, compelling evidence should show the limitations of alternative models in addressing key fundamental or translational inquiries. Moreover, valid findings indicate that the non-human primate model can more effectively guide human applications. Regarding animal ethics and welfare, due to their close phylogenetic relationship with humans, non-human primates are more likely to experience pain, stress, and suffering that is similar to what humans experience. We should consider both their physiological and psychological well-being. Moreover, primates’ complex social groups and dynamics emphasize the need to minimize interference with their social bonding, communication, and emotional health. Additionally, their longer lifespans raise ethical concerns about their long-term welfare and impact on overall health. Although there are unavoidable needs to utilize non-human primate animal models in certain circumstances, the goal is to effectively address significant human health issues while adhering to the principles of the 3Rs (Replacement, Reduction, and Refinement). Furthermore, it is important to foster collaboration, communication, and resource sharing among researchers.

## CONCLUSION AND FUTURE PROSPECTS

In summary, this article offers a comprehensive overview of the embryonic and adult neurogenesis in rodents, monkeys, and humans. We compare the similarities and differences of neurogenesis among these species, highlighting the potential impacts and limitations of non-human primate models on both physiological and pathological neurogenesis research. While neurogenesis in primates is gradually being revealed, we still have several remaining questions that require further investigation. For example, why does neurogenesis in primates occur on a larger scale and take longer during embryonic development compared to rodents? Is this limited neurogenesis in primates solely attributed to the loss of neural stem cell proliferation and differentiation potential, or does it hold a specific evolutionary significance to avoid psychiatric disorders? How can we fully resolve the debates surrounding human adult neurogenesis and understand the significance of its presence or loss? What specific mechanisms govern the fate of neural stem cells in primates? What are their functions under both physiological and pathological conditions?

To address these unknown questions in neurogenesis, the utilization of non-human primate models in combination with gene editing, single-cell multi-omics, imaging and tracing techniques could be powerful. With the continuous advancements in these technologies, we believe that our understanding of primate neurogenesis can be deepened, potentially leading to the development of therapies for a wide range of neural disorders.

## References

[1] Molnár Z , Clowry GJ, Šestan N et al. New insights into the development of the human cerebral cortex. J Anat 2019; 235: 432–51. 10.1111/joa.13055 31373394 PMC6704245

[2] Silbereis JC , Pochareddy S, Zhu Y et al. The cellular and molecular landscapes of the developing Human Central nervous system. Neuron 2016; 89: 248–68. 10.1016/j.neuron.2015.12.008 26796689 PMC4959909

[3] Benito-Kwiecinski S , Giandomenico SL, Sutcliffe M et al. An early cell shape transition drives evolutionary expansion of the human forebrain. Cell 2021; 184: 2084–102. 10.1016/j.cell.2021.02.050 33765444 PMC8054913

[4] Dehay C , Kennedy H, Kosik KS. The outer subventricular zone and primate-specific cortical complexification. Neuron 2015; 85: 683–94. 10.1016/j.neuron.2014.12.060 25695268

[5] Franco SJ , Müller U. Shaping our minds: stem and progenitor cell diversity in the mammalian neocortex. Neuron 2013; 77: 19–34. 10.1016/j.neuron.2012.12.022 23312513 PMC3557841

[6] Hansen DV , Lui JH, Parker PRL et al. Neurogenic radial glia in the outer subventricular zone of human neocortex. Nature 2010; 464: 554–61. 10.1038/nature08845 20154730

[7] Florio M , Huttner WB. Neural progenitors, neurogenesis and the evolution of the neocortex. Development 2014; 141: 2182–94. 10.1242/dev.090571 24866113

[8] Liu J , Liu W, Yang L et al. The primate-specific gene *TMEM14B* marks outer radial glia cells and promotes cortical expansion and folding. Cell Stem Cell 2017; 21: 635–49. 10.1016/j.stem.2017.08.013 29033352

[9] Boldrini M , Fulmore CA, Tartt AN et al. Human hippocampal neurogenesis persists throughout aging. Cell Stem Cell 2018; 22: 589–99. 10.1016/j.stem.2018.03.015 29625071 PMC5957089

[10] Cipriani S , Ferrer I, Aronica E et al. Hippocampal radial glial subtypes and their neurogenic potential in human fetuses and healthy and Alzheimer's Disease adults. Cereb Cortex 2018; 28: 2458–78. 10.1093/cercor/bhy096 29722804

[11] Franjic D , Skarica M, Ma S et al. Transcriptomic taxonomy and neurogenic trajectories of adult human, macaque, and pig hippocampal and entorhinal cells. Neuron 2022; 110: 452–69. 10.1016/j.neuron.2021.10.036 34798047 PMC8813897

[12] Kempermann G , Gage FH, Aigner L et al. Human adult neurogenesis: evidence and remaining questions. Cell Stem Cell 2018; 23: 25–30. 10.1016/j.stem.2018.04.004 29681514 PMC6035081

[13] Moreno-Jiménez EP , Flor-García M, Terreros-Roncal J et al. Adult hippocampal neurogenesis is abundant in neurologically healthy subjects and drops sharply in patients with Alzheimer's disease. Nat Med 2019; 25: 554–60. 10.1038/s41591-019-0375-9 30911133

[14] Sorrells SF , Paredes MF, Cebrian-Silla A et al. Human hippocampal neurogenesis drops sharply in children to undetectable levels in adults. Nature 2018; 555: 377–81. 10.1038/nature25975 29513649 PMC6179355

[15] Terreros-Roncal J , Moreno-Jiménez EP, Flor-García M et al. Impact of neurodegenerative diseases on human adult hippocampal neurogenesis. Science 2021; 374: 1106–13. 10.1126/science.abl5163 34672693 PMC7613437

[16] Tobin MK , Musaraca K, Disouky A et al. Human hippocampal neurogenesis persists in aged adults and Alzheimer's Disease patients. Cell Stem Cell 2019; 24: 974–82. 10.1016/j.stem.2019.05.003 31130513 PMC6608595

[17] Wang W , Wang M, Yang M et al. Transcriptome dynamics of hippocampal neurogenesis in macaques across the lifespan and aged humans. Cell Res 2022; 32: 729–43. 10.1038/s41422-022-00678-y 35750757 PMC9343414

[18] Zhou Y , Su Y, Li S et al. Molecular landscapes of human hippocampal immature neurons across lifespan. Nature 2022; 607: 527–33. 10.1038/s41586-022-04912-w 35794479 PMC9316413

[19] Zhao C , Deng W, Gage FH. Mechanisms and functional implications of adult neurogenesis. Cell 2008; 132: 645–60. 10.1016/j.cell.2008.01.033 18295581

[20] Carletti B , Rossi F. Neurogenesis in the cerebellum. Neuroscientist 2008; 14: 91–100. 10.1177/1073858407304629 17911211

[21] Vanderhaeghen P , Polleux F. Developmental mechanisms underlying the evolution of human cortical circuits. Nat Rev Neurosci 2023; 24: 213–32. 10.1038/s41583-023-00675-z 36792753 PMC10064077

[22] Chédotal A . Roles of axon guidance molecules in neuronal wiring in the developing spinal cord. Nat Rev Neurosci 2019; 20: 380–96. 10.1038/s41583-019-0168-7 31000796

[23] Aida T , Feng G. The dawn of non-human primate models for neurodevelopmental disorders. Curr Opin Genet Dev 2020; 65: 160–8. 10.1016/j.gde.2020.05.040 32693220 PMC7955645

[24] Bystron I , Blakemore C, Rakic P. Development of the human cerebral cortex: boulder Committee revisited. Nat Rev Neurosci 2008; 9: 110–22. 10.1038/nrn2252 18209730

[25] Sauer FC . Mitosis in the neural tube. J Comp Neurol 1935; 62: 377–405.

[26] Rakic P . Specification of cerebral cortical areas. Science 1988; 241: 170–6. 10.1126/science.3291116 3291116

[27] Sidman RL , Rakic P. Neuronal migration, with special reference to developing human brain: a review. Brain Res 1973; 62: 1–35. 10.1016/0006-8993(73)90617-3 4203033

[28] Götz M , Huttner WB. The cell biology of neurogenesis. Nat Rev Mol Cell Biol 2005; 6: 777–88. 10.1038/nrm1739 16314867

[29] Smart IH . Proliferative characteristics of the ependymal layer during the early development of the mouse neocortex: a pilot study based on recording the number, location and plane of cleavage of mitotic figures. J Anat 1973; 116: 67–91. 4777782 PMC1271551

[30] Miller DJ , Bhaduri A, Sestan N et al. Shared and derived features of cellular diversity in the human cerebral cortex. Curr Opin Neurobiol 2019; 56: 117–24. 10.1016/j.conb.2018.12.005 30677551 PMC6996583

[31] Haubensak W , Attardo A, Denk W et al. Neurons arise in the basal neuroepithelium of the early mammalian telencephalon: a major site of neurogenesis. Proc Natl Acad Sci USA 2004; 101: 3196–201. 10.1073/pnas.0308600100 14963232 PMC365766

[32] Noctor SC , Martínez-Cerdeño V, Ivic L et al. Cortical neurons arise in symmetric and asymmetric division zones and migrate through specific phases. Nat Neurosci 2004; 7: 136–44. 10.1038/nn1172 14703572

[33] Betizeau M , Cortay V, Patti D et al. Precursor diversity and complexity of lineage relationships in the outer subventricular zone of the primate. Neuron 2013; 80: 442–57. 10.1016/j.neuron.2013.09.032 24139044

[34] Fietz SA , Kelava I, Vogt J et al. OSVZ progenitors of human and ferret neocortex are epithelial-like and expand by integrin signaling. Nat Neurosci 2010; 13: 690–9. 10.1038/nn.2553 20436478

[35] Wang X , Tsai J-W, Lamonica B et al. A new subtype of progenitor cell in the mouse embryonic neocortex. Nat Neurosci 2011; 14: 555–61. 10.1038/nn.2807 21478886 PMC3083489

[36] Huttner WB , Kosodo Y. Symmetric versus asymmetric cell division during neurogenesis in the developing vertebrate central nervous system. Curr Opin Cell Biol 2005; 17: 648–57. 10.1016/j.ceb.2005.10.005 16243506

[37] Lui JH , Hansen DV, Kriegstein AR. Development and evolution of the human neocortex. Cell 2011; 146: 18–36. 10.1016/j.cell.2011.06.030 21729779 PMC3610574

[38] Kelley KW , Pașca SP. Human brain organogenesis: toward a cellular understanding of development and disease. Cell 2022; 185: 42–61. 10.1016/j.cell.2021.10.003 34774127 PMC13523617

[39] Bae B-I , Jayaraman D, Walsh CA. Genetic changes shaping the human brain. Dev Cell 2015; 32: 423–34. 10.1016/j.devcel.2015.01.035 25710529 PMC4429600

[40] Dehay C , Kennedy H. Cell-cycle control and cortical development. Nat Rev Neurosci 2007; 8: 438–50. 10.1038/nrn2097 17514197

[41] Smart IHM , Dehay C, Giroud P et al. Unique morphological features of the proliferative zones and postmitotic compartments of the neural epithelium giving rise to striate and extrastriate cortex in the monkey. Cereb Cortex 2002; 12: 37–53. 10.1093/cercor/12.1.37 11734531 PMC1931430

[42] Nowakowski TJ , Pollen AA, Sandoval-Espinosa C et al. Transformation of the radial glia scaffold demarcates two stages of Human cerebral cortex development. Neuron 2016; 91: 1219–27. 10.1016/j.neuron.2016.09.005 27657449 PMC5087333

[43] de Azevedo LC , Fallet C, Moura-Neto V et al. Cortical radial glial cells in human fetuses: depth-correlated transformation into astrocytes. J Neurobiol 2003; 55: 288–98. 10.1002/neu.10205 12717699

[44] Yang L , Li Z, Liu G et al. Developmental origins of Human cortical oligodendrocytes and astrocytes. Neurosci Bull 2022; 38: 47–68. 10.1007/s12264-021-00759-9 34374948 PMC8783027

[45] Schmechel DE , Rakic P. Arrested proliferation of radial glial cells during midgestation in rhesus monkey. Nature 1979; 277: 303–5. 10.1038/277303a0 105294

[46] Iacopetti P , Michelini M, Stuckmann I et al. Expression of the antiproliferative gene TIS21 at the onset of neurogenesis identifies single neuroepithelial cells that switch from proliferative to neuron-generating division. Proc Natl Acad Sci USA 1999; 96: 4639–44. 10.1073/pnas.96.8.4639 10200315 PMC16385

[47] Chenn A , McConnell SK. Cleavage orientation and the asymmetric inheritance of Notch1 immunoreactivity in mammalian neurogenesis. Cell 1995; 82: 631–41. 10.1016/0092-8674(95)90035-7 7664342

[48] Marín-Padilla M . The mammalian neocortex new pyramidal neuron: a new conception. Front Neuroanat 2014; 7: 51. 10.3389/fnana.2013.00051 24431992 PMC3880895

[49] Bystron I , Rakic P, Molnár Z et al. The first neurons of the human cerebral cortex. Nat Neurosci 2006; 9: 880–6. 10.1038/nn1726 16783367

[50] García-Moreno F , López-Mascaraque L, De Carlos JA. Origins and migratory routes of murine Cajal-Retzius cells. J Comp Neurol 2007; 500: 419–32. 10.1002/cne.21128 17120279

[51] Takahashi T , Goto T, Miyama S et al. Sequence of neuron origin and neocortical laminar fate: relation to cell cycle of origin in the developing murine cerebral wall. J Neurosci 1999; 19: 10357–71. 10.1523/JNEUROSCI.19-23-10357.1999 10575033 PMC6782435

[52] Li J , Sun L, Peng X-L et al. Integrative genomic analysis of early neurogenesis reveals a temporal genetic program for differentiation and specification of preplate and Cajal-Retzius neurons. PLoS Genet 2021; 17: e1009355. 10.1371/journal.pgen.1009355 33760820 PMC7990179

[53] Rakic P . Prenatal development of the visual system in rhesus monkey. Philos Trans R Soc Lond B Biol Sci 1977; 278: 245–60. 19781 10.1098/rstb.1977.0040

[54] Hoerder-Suabedissen A , Molnár Z. Development, evolution and pathology of neocortical subplate neurons. Nat Rev Neurosci 2015; 16: 133–46. 10.1038/nrn3915 25697157

[55] Kanold PO , Luhmann HJ. The subplate and early cortical circuits. Annu Rev Neurosci 2010; 33: 23–48. 10.1146/annurev-neuro-060909-153244 20201645

[56] Molnár Z , Luhmann HJ, Kanold PO. Transient cortical circuits match spontaneous and sensory-driven activity during development. Science 2020; 370. 10.1126/science.abb2153 PMC8050953 33060328

[57] Duque A , Krsnik Z, Kostović I et al. Secondary expansion of the transient subplate zone in the developing cerebrum of human and nonhuman primates. Proc Natl Acad Sci USA 2016; 113: 9892–7. 10.1073/pnas.1610078113 27503885 PMC5024589

[58] Rakic P . Neurons in rhesus monkey visual cortex: systematic relation between time of origin and eventual disposition. Science 1974; 183: 425–7. 10.1126/science.183.4123.425 4203022

[59] Angevine JB , Sidman RL. Autoradiographic study of cell migration during histogenesis of cerebral cortex in the mouse. Nature 1961; 192: 766–8. 10.1038/192766b0 17533671

[60] Cadwell CR , Bhaduri A, Mostajo-Radji MA et al. Development and arealization of the cerebral cortex. Neuron 2019; 103: 980–1004. 10.1016/j.neuron.2019.07.009 31557462 PMC9245854

[61] Balaram P , Kaas J, Young N. Histological features of layers and sublayers in cortical visual areas V1 and V2 of chimpanzees, macaque monkeys, and humans. Eye Brain 2014; 2014: 5–18. 10.2147/EB.S51814 25788835 PMC4360995

[62] Levitt P , Cooper ML, Rakic P. Early divergence and changing proportions of neuronal and glial precursor cells in the primate cerebral ventricular zone. Dev Biol 1983; 96: 472–84. 10.1016/0012-1606(83)90184-7 6339301

[63] Jakovcevski I , Filipovic R, Mo Z et al. Oligodendrocyte development and the onset of myelination in the human fetal brain. Front Neuroanat 2009; 3: 5. 10.3389/neuro.05.005.2009 19521542 PMC2694674

[64] Yeung MSY , Zdunek S, Bergmann O et al. Dynamics of oligodendrocyte generation and myelination in the human brain. Cell 2014; 159: 766–74. 10.1016/j.cell.2014.10.011 25417154

[65] Zhu Y , Sousa AMM, Gao T et al. Spatiotemporal transcriptomic divergence across human and macaque brain development. Science 2018; 362: eaat8077. 10.1126/science.aat8077 30545855 PMC6900982

[66] Rakic P . A small step for the cell, a giant leap for mankind: a hypothesis of neocortical expansion during evolution. Trends Neurosci 1995; 18: 383–8. 10.1016/0166-2236(95)93934-P 7482803

[67] Rakic P . Neurogenesis in adult primate neocortex: an evaluation of the evidence. Nat Rev Neurosci 2002; 3: 65–71. 10.1038/nrn700 11823806

[68] Rash BG , Duque A, Morozov YM et al. Gliogenesis in the outer subventricular zone promotes enlargement and gyrification of the primate cerebrum. Proc Natl Acad Sci USA 2019; 116: 7089–94. 10.1073/pnas.1822169116 30894491 PMC6452694

[69] Huang W , Bhaduri A, Velmeshev D et al. Origins and proliferative states of Human oligodendrocyte precursor cells. Cell 2020; 182: 594–608. 10.1016/j.cell.2020.06.027 32679030 PMC7415734

[70] Ma S , Skarica M, Li Q et al. Molecular and cellular evolution of the primate dorsolateral prefrontal cortex. Science 2022; 377: eabo7257. 10.1126/science.abo7257 36007006 PMC9614553

[71] Herculano-Houzel S . The remarkable, yet not extraordinary, human brain as a scaled-up primate brain and its associated cost. Proc Natl Acad Sci USA 2012; 109 Suppl 1: 10661–8. 10.1073/pnas.1201895109 22723358 PMC3386878

[72] Herculano-Houzel S . The human brain in numbers: a linearly scaled-up primate brain. Front Hum Neurosci 2009; 3: 31. 10.3389/neuro.09.031.2009 19915731 PMC2776484

[73] Libé-Philippot B , Vanderhaeghen P. Cellular and molecular mechanisms linking Human cortical development and evolution. Annu Rev Genet 2021; 55: 555–81. 10.1146/annurev-genet-071719-020705 34535062

[74] Polleux F , Dehay C, Moraillon B et al. Regulation of neuroblast cell-cycle kinetics plays a crucial role in the generation of unique features of neocortical areas. J Neurosci 1997; 17: 7763–83. 10.1523/JNEUROSCI.17-20-07763.1997 9315898 PMC6793912

[75] Kornack DR , Rakic P. Changes in cell-cycle kinetics during the development and evolution of primate neocortex. Proc Natl Acad Sci USA 1998; 95: 1242–6. 10.1073/pnas.95.3.1242 9448316 PMC18732

[76] Iwata R , Casimir P, Vanderhaeghen P. Mitochondrial dynamics in postmitotic cells regulate neurogenesis. Science 2020; 369: 858–62. 10.1126/science.aba9760 32792401

[77] Bayer SA, Altman J and Russo RJ et al. Timetables of neurogenesis in the human brain based on experimentally determined patterns in the rat. Neurotoxicology 1993; 14: 83–144. 8361683

[78] Rakic P . The radial edifice of cortical architecture: from neuronal silhouettes to genetic engineering. Brain Res Rev 2007; 55: 204–19. 10.1016/j.brainresrev.2007.02.010 17467805 PMC2203611

[79] Zecevic N , Chen Y, Filipovic R. Contributions of cortical subventricular zone to the development of the human cerebral cortex. J Comp Neurol 2005; 491: 109–22. 10.1002/cne.20714 16127688 PMC2628573

[80] Kriegstein A , Noctor S, Martínez-Cerdeño V. Patterns of neural stem and progenitor cell division may underlie evolutionary cortical expansion. Nat Rev Neurosci 2006; 7: 883–90. 10.1038/nrn2008 17033683

[81] Wu S-X , Goebbels S, Nakamura K et al. Pyramidal neurons of upper cortical layers generated by NEX-positive progenitor cells in the subventricular zone. Proc Natl Acad Sci USA 2005; 102: 17172–7. 10.1073/pnas.0508560102 16284248 PMC1288007

[82] Martínez-Cerdeño V , Cunningham CL, Camacho J et al. Comparative analysis of the subventricular zone in rat, ferret and macaque: evidence for an outer subventricular zone in rodents. PLoS One 2012; 7: e30178. 10.1371/journal.pone.0030178 22272298 PMC3260244

[83] Shitamukai A , Konno D, Matsuzaki F. Oblique radial glial divisions in the developing mouse neocortex induce self-renewing progenitors outside the germinal zone that resemble primate outer subventricular zone progenitors. J Neurosci 2011; 31: 3683–95. 10.1523/JNEUROSCI.4773-10.2011 21389223 PMC6622781

[84] Pollen AA , Nowakowski TJ, Chen J et al. Molecular identity of human outer radial glia during cortical development. Cell 2015; 163: 55–67. 10.1016/j.cell.2015.09.004 26406371 PMC4583716

[85] Eze UC , Bhaduri A, Haeussler M et al. Single-cell atlas of early human brain development highlights heterogeneity of human neuroepithelial cells and early radial glia. Nat Neurosci 2021; 24: 584–94. 10.1038/s41593-020-00794-1 33723434 PMC8012207

[86] Zeng B , Liu Z, Lu Y et al. The single-cell and spatial transcriptional landscape of human gastrulation and early brain development. Cell Stem Cell 2023; **30**: 851–66. 10.1016/j.stem.2023.04.016 PMC10241223 37192616

[87] BRAIN Initiative Cell Census Network . A multimodal cell census and atlas of the mammalian primary motor cortex. Nature 2021; 598: 86–102. https://www.nature.com/articles/s41586-021-03950-0 34616075 10.1038/s41586-021-03950-0 PMC8494634

[88] Bakken TE , Jorstad NL, Hu Q et al. Comparative cellular analysis of motor cortex in human, marmoset and mouse. Nature 2021; 598: 111–9. 10.1038/s41586-021-03465-8 34616062 PMC8494640

[89] Khrameeva E , Kurochkin I, Han D et al. Single-cell-resolution transcriptome map of human, chimpanzee, bonobo, and macaque brains. Genome Res 2020; 30: 776–89. 10.1101/gr.256958.119 32424074 PMC7263190

[90] Chen A , Sun Y, Lei Y et al. Single-cell spatial transcriptome reveals cell-type organization in the macaque cortex. Cell 2023; **186**: 3726–43. 10.1016/j.cell.2023.06.009 37442136

[91] Berg J , Sorensen SA, Ting JT et al. Human neocortical expansion involves glutamatergic neuron diversification. Nature 2021; 598: 151–8. 10.1038/s41586-021-03813-8 34616067 PMC8494638

[92] Shi Y , Wang M, Mi D et al. Mouse and human share conserved transcriptional programs for interneuron development. Science 2021; 374: eabj6641. 10.1126/science.abj6641 34882453 PMC7618238

[93] Fertuzinhos S , Krsnik Ž, Kawasawa YI et al. Selective depletion of molecularly defined cortical interneurons in human holoprosencephaly with severe striatal hypoplasia. Cereb Cortex 2009; 19: 2196–207. 10.1093/cercor/bhp009 19234067 PMC2722430

[94] Letinic K , Zoncu R, Rakic P. Origin of GABAergic neurons in the human neocortex. Nature 2002; 417: 645–9. 10.1038/nature00779 12050665

[95] Petanjek Z , Berger B, Esclapez M. Origins of cortical GABAergic neurons in the cynomolgus monkey. Cereb Cortex 2009; 19: 249–62. 10.1093/cercor/bhn078 18477686 PMC2638783

[96] Zecevic N , Hu F, Jakovcevski I. Interneurons in the developing human neocortex. Dev Neurobiol 2011; 71: 18–33. 10.1002/dneu.20812 21154907 PMC3117059

[97] Hladnik A , Džaja D, Darmopil S et al. Spatio-temporal extension in site of origin for cortical calretinin neurons in primates. Front Neuroanat 2014; 8: 50. 10.3389/fnana.2014.00050 25018702 PMC4072090

[98] Yu X , Zecevic N. Dorsal radial glial cells have the potential to generate cortical interneurons in human but not in mouse brain. J Neurosci 2011; 31: 2413–20. 10.1523/JNEUROSCI.5249-10.2011 21325508 PMC3079257

[99] Delgado RN , Allen DE, Keefe MG et al. Individual human cortical progenitors can produce excitatory and inhibitory neurons. Nature 2022; 601: 397–403. 10.1038/s41586-021-04230-7 34912114 PMC8994470

[100] Polleux F, Dehay C and Goffinet A . Pre- and post-mitotic events contribute to the progressive acquisition of area-specific connectional fate in the neocortex. Cereb Cortex 2001; 11: 1027–39. 10.1093/cercor/11.11.1027 11590112

[101] Seuntjens E , Nityanandam A, Miquelajauregui A et al. Sip1 regulates sequential fate decisions by feedback signaling from postmitotic neurons to progenitors. Nat Neurosci 2009; 12: 1373–80. 10.1038/nn.2409 19838179

[102] Fietz SA , Lachmann R, Brandl H et al. Transcriptomes of germinal zones of human and mouse fetal neocortex suggest a role of extracellular matrix in progenitor self-renewal. Proc Natl Acad Sci USA 2012; 109: 11836–41. 10.1073/pnas.1209647109 22753484 PMC3406833

[103] Dehay C , Savatier P, Cortay V et al. Cell-cycle kinetics of neocortical precursors are influenced by embryonic thalamic axons. J Neurosci 2001; 21: 201–14. 10.1523/JNEUROSCI.21-01-00201.2001 11150337 PMC6762433

[104] Monko T , Rebertus J, Stolley J et al. Thalamocortical axons regulate neurogenesis and laminar fates in the early sensory cortex. Proc Natl Acad Sci USA 2022; 119: e2201355119. 10.1073/pnas.2201355119 35613048 PMC9295754

[105] Marin-Padilla M . Structural organization of the human cerebral cortex prior to the appearance of the cortical plate. Anat Embryol (Berl) 1983; 168: 21–40. 10.1007/BF00305396 6650855

[106] Žunić Išasegi I , Radoš M, Krsnik Ž et al. Interactive histogenesis of axonal strata and proliferative zones in the human fetal cerebral wall. Brain Struct Funct 2018; 223: 3919–43. 10.1007/s00429-018-1721-2 30094607 PMC6267252

[107] Zecevic N , Rakic P. Development of layer I neurons in the primate cerebral cortex. J Neurosci 2001; 21: 5607–19. 10.1523/JNEUROSCI.21-15-05607.2001 11466432 PMC6762645

[108] Meyer G , González-Gómez M. The subpial granular layer and transient versus persisting Cajal-Retzius neurons of the fetal Human cortex. Cereb Cortex 2018; 28: 2043–58. 10.1093/cercor/bhx110 28472243

[109] Causeret F , Moreau MX, Pierani A et al. The multiple facets of Cajal-Retzius neurons. Development 2021; 148: dev199409. 10.1242/dev.199409 34047341

[110] Sousa AMM , Meyer KA, Santpere G et al. Evolution of the Human nervous system function, structure, and development. Cell 2017; 170: 226–47. 10.1016/j.cell.2017.06.036 28708995 PMC5647789

[111] Espinós A , Fernández‐Ortuño E, Negri E et al. Evolution of genetic mechanisms regulating cortical neurogenesis. Dev Neurobiol 2022; 82: 428–53. 10.1002/dneu.22891 35670518 PMC9543202

[112] Zhu X , Guo Y, Chu C et al. *BRN_2_* as a key gene drives the early primate telencephalon development. Sci Adv 2022; 8: eabl7263. 10.1126/sciadv.abl7263 35245119 PMC8896791

[113] Brattås PL , Jönsson ME, Fasching L et al. TRIM_2_8 Controls a gene regulatory network based on endogenous retroviruses in Human neural progenitor cells. Cell Rep 2017; 18: 1–11. 10.1016/j.celrep.2016.12.010 28052240

[114] Takahashi K , Jeong D, Wang S et al. Critical roles of translation initiation and RNA uridylation in endogenous retroviral expression and neural differentiation in pluripotent stem cells. Cell Rep 2020; 31: 107715. 10.1016/j.celrep.2020.107715 32492424 PMC8195978

[115] Wang T , Medynets M, Johnson KR et al. Regulation of stem cell function and neuronal differentiation by HERV-K via mTOR pathway. Proc Natl Acad Sci USA 2020; 117: 17842–53. 10.1073/pnas.2002427117 32669437 PMC7395438

[116] Keeney JG , Davis JM, Siegenthaler J et al. DUF1220 protein domains drive proliferation in human neural stem cells and are associated with increased cortical volume in anthropoid primates. Brain Struct Funct 2015; 220: 3053–60. 10.1007/s00429-014-0814-9 24957859 PMC4722867

[117] Marques-Bonet T , Eichler EE. The evolution of human segmental duplications and the core duplicon hypothesis. Cold Spring Harb Symp Quant Biol 2009; 74: 355–62. 10.1101/sqb.2009.74.011 19717539 PMC4114149

[118] MacDermot KD , Bonora E, Sykes N et al. Identification of FOXP2 truncation as a novel cause of developmental speech and language deficits. Am J Hum Genet 2005; 76: 1074–80. 10.1086/430841 15877281 PMC1196445

[119] Enard W , Gehre S, Hammerschmidt K et al. A humanized version of Foxp2 affects cortico-basal ganglia circuits in mice. Cell 2009; 137: 961–71. 10.1016/j.cell.2009.03.041 19490899

[120] Tsui D , Vessey JP, Tomita H et al. FoxP2 regulates neurogenesis during embryonic cortical development. J Neurosci 2013; 33: 244–58. 10.1523/JNEUROSCI.1665-12.2013 23283338 PMC6618635

[121] Pinson A , Xing L, Namba T et al. Human TKTL1 implies greater neurogenesis in frontal neocortex of modern humans than Neanderthals. Science 2022; 377: eabl6422. 10.1126/science.abl6422 36074851

[122] Kaessmann H . Origins, evolution, and phenotypic impact of new genes. Genome Res 2010; 20: 1313–26. 10.1101/gr.101386.109 20651121 PMC2945180

[123] Florio M , Albert M, Taverna E et al. Human-specific gene *ARHGAP11B* promotes basal progenitor amplification and neocortex expansion. Science 2015; 347: 1465–70. 10.1126/science.aaa1975 25721503

[124] Hou Q-Q , Xiao Q, Sun X-Y et al. TBC1D3 promotes neural progenitor proliferation by suppressing the histone methyltransferase G9a. Sci Adv 2021; 7: eaba8053. 10.1126/sciadv.aba8053 PMC7810367 33523893

[125] Suzuki IK , Gacquer D, Van Heurck R et al. Human-specific *NOTCH2NL* genes expand cortical neurogenesis through delta/notch regulation. Cell 2018; 173: 1370–84. 10.1016/j.cell.2018.03.067 29856955 PMC6092419

[126] Van Heurck R , Bonnefont J, Wojno M et al. CROCCP2 acts as a human-specific modifier of cilia dynamics and mTOR signaling to promote expansion of cortical progenitors. Neuron 2023; 111: 65–80. 10.1016/j.neuron.2022.10.018 36334595 PMC9831670

[127] Heide M , Haffner C, Murayama A et al. Human-specific *ARHGAP_11_B* increases size and folding of primate neocortex in the fetal marmoset. Science 2020; 369: 546–50. 10.1126/science.abb2401 32554627

[128] Kalebic N , Gilardi C, Albert M et al. Human-specific *ARHGAP_11_B* induces hallmarks of neocortical expansion in developing ferret neocortex. Elife 2018; 7: e41241. 10.7554/eLife.41241 PMC6303107 30484771

[129] Ju X-C , Hou Q-Q, Sheng A-L et al. The hominoid-specific gene *TBC1D3* promotes generation of basal neural progenitors and induces cortical folding in mice. Elife 2016; 5: e18197. 10.7554/eLife.18197 PMC5028191 27504805

[130] Iwata R , Casimir P, Erkol E et al. Mitochondria metabolism sets the species-specific tempo of neuronal development. Science 2023; 379: eabn4705. 10.1126/science.abn4705 36705539

[131] Rayon T , Stamataki D, Perez-Carrasco R et al. Species-specific pace of development is associated with differences in protein stability. Science 2020; 369: eaba7667. 10.1126/science.aba7667 PMC7116327 32943498

[132] Ba Q , Hei Y, Dighe A et al. Proteotype coevolution and quantitative diversity across 11 mammalian species. Sci Adv 2022; 8: eabn0756. 10.1126/sciadv.abn0756 36083897 PMC9462687

[133] Hoye ML , Calviello L, Poff AJ et al. Aberrant cortical development is driven by impaired cell cycle and translational control in a *DDX3X* syndrome model. Elife 2022; 11: e78203. 10.7554/eLife.78203 PMC9239684 35762573

[134] Wu Q , Shichino Y, Abe T et al. Selective translation of epigenetic modifiers affects the temporal pattern and differentiation of neural stem cells. Nat Commun 2022; 13: 470. 10.1038/s41467-022-28097-y 35078993 PMC8789897

[135] Penisson M , Ladewig J, Belvindrah R et al. Genes and mechanisms involved in the generation and amplification of basal radial glial cells. Front Cell Neurosci 2019; 13: 381. 10.3389/fncel.2019.00381 31481878 PMC6710321

[136] Geschwind DH , Rakic P. Cortical evolution: judge the brain by its cover. Neuron 2013; 80: 633–47. 10.1016/j.neuron.2013.10.045 24183016 PMC3922239

[137] Trujillo CA , Muotri AR. Brain organoids and the study of neurodevelopment. Trends Mol Med 2018; 24: 982–90. 10.1016/j.molmed.2018.09.005 30377071 PMC6289846

[138] Guy J , Hendrich B, Holmes M et al. A mouse *Mecp2*-null mutation causes neurological symptoms that mimic Rett syndrome. Nat Genet 2001; 27: 322–6. 10.1038/85899 11242117

[139] Chen Y , Yu J, Niu Y et al. Modeling Rett syndrome using TALEN-edited *MECP2* mutant cynomolgus monkeys. Cell 2017; 169: 945–55. 10.1016/j.cell.2017.04.035 28525759 PMC5540256

[140] Wang X , Bey AL, Katz BM et al. Altered mGluR5-Homer scaffolds and corticostriatal connectivity in a *Shank3* complete knockout model of autism. Nat Commun 2016; 7: 11459. 10.1038/ncomms11459 27161151 PMC4866051

[141] Zhou Y , Kaiser T, Monteiro P et al. Mice with *Shank3* mutations associated with ASD and Schizophrenia display both shared and distinct defects. Neuron 2016; 89: 147–62. 10.1016/j.neuron.2015.11.023 26687841 PMC4754122

[142] Zhou Y , Sharma J, Ke Q et al. Atypical behaviour and connectivity in *SHANK3*-mutant macaques. Nature 2019; 570: 326–31. 10.1038/s41586-019-1278-0 31189958

[143] Li B , Zhao H, Tu Z et al. *CHD8* mutations increase gliogenesis to enlarge brain size in the nonhuman primate. Cell Discov 2023; 9: 27. 10.1038/s41421-023-00525-3 36878905 PMC9988832

[144] Gruber R , Zhou Z, Sukchev M et al. MCPH1 regulates the neuroprogenitor division mode by coupling the centrosomal cycle with mitotic entry through the Chk1-Cdc25 pathway. Nat Cell Biol 2011; 13: 1325–34. 10.1038/ncb2342 21947081

[145] Chen J , Ingham N, Clare S et al. *Mcph1*-deficient mice reveal a role for MCPH1 in otitis media. PLoS One 2013; 8: e58156. 10.1371/journal.pone.0058156 23516444 PMC3596415

[146] Trimborn M , Ghani M, Walther DJ et al. Establishment of a mouse model with misregulated chromosome condensation due to defective Mcph1 function. PLoS One 2010; 5: e9242. 10.1371/journal.pone.0009242 20169082 PMC2821930

[147] Liang Y , Gao H, Lin S-Y et al. BRIT1/MCPH1 is essential for mitotic and meiotic recombination DNA repair and maintaining genomic stability in mice. PLoS Genet 2010; 6: e1000826. 10.1371/journal.pgen.1000826 20107607 PMC2809772

[148] Ke Q , Li W, Lai X et al. TALEN-based generation of a cynomolgus monkey disease model for human microcephaly. Cell Res 2016; 26: 1048–61. 10.1038/cr.2016.93 27502025 PMC5034111

[149] Vallee RB , Tsai J-W. The cellular roles of the lissencephaly gene LIS1, and what they tell us about brain development. Genes Dev 2006; 20: 1384–93. 10.1101/gad.1417206 16751177

[150] Gould E , Gross CG. Neurogenesis in adult mammals: some progress and problems. J Neurosci 2002; 22: 619–23. 10.1523/JNEUROSCI.22-03-00619.2002 11826089 PMC6758509

[151] Alvarez-Buylla A , Lim DA. For the long run: maintaining germinal niches in the adult brain. Neuron 2004; 41: 683–6. 10.1016/S0896-6273(04)00111-4 15003168

[152] Kempermann G . New neurons for ‘survival of the fittest.’. Nat Rev Neurosci 2012; 13: 727–36. 10.1038/nrn3319 22948073

[153] Paredes MF , Sorrells SF, Garcia‐Verdugo JM et al. Brain size and limits to adult neurogenesis. J Comp Neurol 2016; 524: 646–64. 10.1002/cne.23896 26417888 PMC5047485

[154] Altman J . Autoradiographic investigation of cell proliferation in the brains of rats and cats. Anat Rec 1963; 145: 573–91. 10.1002/ar.1091450409 14012334

[155] Altman J , Das GD. Autoradiographic and histological evidence of postnatal hippocampal neurogenesis in rats. J Comp Neurol 1965; 124: 319–35. 10.1002/cne.901240303 5861717

[156] Seki T , Arai Y. Highly polysialylated neural cell adhesion molecule (NCAM-H) is expressed by newly generated granule cells in the dentate gyrus of the adult rat. J Neurosci 1993; 13: 2351–8. 10.1523/JNEUROSCI.13-06-02351.1993 7684771 PMC6576495

[157] Seki T , Arai Y. Age-related production of new granule cells in the adult dentate gyrus. Neuroreport 1995; 6: 2479–82. 10.1097/00001756-199512150-00010 8741746

[158] Kuhn HG , Dickinson-Anson H, Gage F. Neurogenesis in the dentate gyrus of the adult rat: age-related decrease of neuronal progenitor proliferation. J Neurosci 1996; 16: 2027–33. 10.1523/JNEUROSCI.16-06-02027.1996 8604047 PMC6578509

[159] Ming G-L , Song H. Adult neurogenesis in the mammalian brain: significant answers and significant questions. Neuron 2011; 70: 687–702. 10.1016/j.neuron.2011.05.001 21609825 PMC3106107

[160] Kempermann G , Song H, Gage FH. Neurogenesis in the adult hippocampus. Cold Spring Harb Perspect Biol 2015; 7: a018812. 10.1101/cshperspect.a018812 26330519 PMC4563705

[161] Bond AM , Ming G-L, Song H. Adult mammalian neural stem cells and neurogenesis: five decades later. Cell Stem Cell 2015; 17: 385–95. 10.1016/j.stem.2015.09.003 26431181 PMC4683085

[162] Pellegrino G , Trubert C, Terrien J et al. A comparative study of the neural stem cell niche in the adult hypothalamus of human, mouse, rat and gray mouse lemur (Microcebus murinus). J Comp Neurol 2018; 526: 1419–43. 10.1002/cne.24376 29230807

[163] Jurkowski MP , Bettio L, K. Woo E et al. Beyond the hippocampus and the SVZ: adult neurogenesis throughout the brain. Front Cell Neurosci 2020; 14: 576444. 10.3389/fncel.2020.576444 33132848 PMC7550688

[164] Lee SW , Clemenson GD, Gage FH. New neurons in an aged brain. Behav Brain Res 2012; 227: 497–507. 10.1016/j.bbr.2011.10.009 22024433 PMC3264739

[165] Semerci F , Maletic-Savatic M. Transgenic mouse models for studying adult neurogenesis. Front Biol (Beijing) 2016; 11: 151–67. 10.1007/s11515-016-1405-3 28473846 PMC5412727

[166] Hochgerner H , Zeisel A, Lönnerberg P et al. Conserved properties of dentate gyrus neurogenesis across postnatal development revealed by single-cell RNA sequencing. Nat Neurosci 2018; 21: 290–9. 10.1038/s41593-017-0056-2 29335606

[167] Gould E , Reeves AJ, Fallah M et al. Hippocampal neurogenesis in adult Old World primates. Proc Natl Acad Sci USA 1999; 96: 5263–7. 10.1073/pnas.96.9.5263 10220454 PMC21852

[168] Kornack DR , Rakic P. Continuation of neurogenesis in the hippocampus of the adult macaque monkey. Proc Natl Acad Sci USA 1999; 96: 5768–73. 10.1073/pnas.96.10.5768 10318959 PMC21935

[169] Sawamoto K , Hirota Y, Alfaro-Cervello C et al. Cellular composition and organization of the subventricular zone and rostral migratory stream in the adult and neonatal common marmoset brain. J Comp Neurol 2011; 519: 690–713. 10.1002/cne.22543 21246550 PMC4096931

[170] Kornack DR , Rakic P. Cell proliferation without neurogenesis in adult primate neocortex. Science 2001; 294: 2127–30. 10.1126/science.1065467 11739948

[171] Bernier PJ , Bedard A, Vinet J et al. Newly generated neurons in the amygdala and adjoining cortex of adult primates. Proc Natl Acad Sci USA 2002; 99: 11464–9. 10.1073/pnas.172403999 12177450 PMC123279

[172] Gould E , Vail N, Wagers M et al. Adult-generated hippocampal and neocortical neurons in macaques have a transient existence. Proc Natl Acad Sci USA 2001; 98: 10910–7. 10.1073/pnas.181354698 11526209 PMC58573

[173] Seki T . Understanding the real State of Human adult hippocampal neurogenesis from studies of rodents and non-human primates. Front Neurosci 2020; 14: 839. 10.3389/fnins.2020.00839 32848586 PMC7432251

[174] Li Y , Xu N-N, Hao Z-Z et al. Adult neurogenesis in the primate hippocampus. Zool Res 2023; 44: 315–22. 10.24272/j.issn.2095-8137.2022.399 36785898 PMC10083228

[175] Gould E , McEwen BS, Tanapat P et al. Neurogenesis in the dentate gyrus of the adult tree shrew is regulated by psychosocial stress and NMDA receptor activation. J Neurosci 1997; 17: 2492–8. 10.1523/JNEUROSCI.17-07-02492.1997 9065509 PMC6573503

[176] Gould E , Tanapat P, McEwen BS et al. Proliferation of granule cell precursors in the dentate gyrus of adult monkeys is diminished by stress. Proc Natl Acad Sci USA 1998; 95: 3168–71. 10.1073/pnas.95.6.3168 9501234 PMC19713

[177] Perera TD , Coplan JD, Lisanby SH et al. Antidepressant-induced neurogenesis in the hippocampus of adult nonhuman primates. J Neurosci 2007; 27: 4894–901. 10.1523/JNEUROSCI.0237-07.2007 17475797 PMC6672102

[178] Koketsu D , Furuichi Y, Maeda M et al. Increased number of new neurons in the olfactory bulb and hippocampus of adult non-human primates after focal ischemia. Exp Neurol 2006; 199: 92–102. 10.1016/j.expneurol.2006.03.012 16712840

[179] Tonchev AB , Yamashima T, Zhao L et al. Proliferation of neural and neuronal progenitors after global brain ischemia in young adult macaque monkeys. Mol Cell Neurosci 2003; 23: 292–301. 10.1016/S1044-7431(03)00058-7 12812760

[180] Yamashima T , Tonchev AB, Vachkov IH et al. Vascular adventitia generates neuronal progenitors in the monkey hippocampus after ischemia. Hippocampus 2004; 14: 861–75. 10.1002/hipo.20001 15382256

[181] Hao Z-Z , Wei J-R, Xiao D et al. Single-cell transcriptomics of adult macaque hippocampus reveals neural precursor cell populations. Nat Neurosci 2022; 25: 805–17. 10.1038/s41593-022-01073-x 35637371

[182] Zhang H , Li J, Ren J et al. Single-nucleus transcriptomic landscape of primate hippocampal aging. Protein Cell 2021; 12: 695–716. 10.1007/s13238-021-00852-9 34052996 PMC8403220

[183] Kohler SJ , Williams NI, Stanton GB et al. Maturation time of new granule cells in the dentate gyrus of adult macaque monkeys exceeds six months. Proc Natl Acad Sci USA 2011; 108: 10326–31. 10.1073/pnas.1017099108 21646517 PMC3121825

[184] Aizawa K , Ageyama N, Yokoyama C et al. Age-dependent alteration in hippocampal neurogenesis correlates with learning performance of macaque monkeys. Exp Anim 2009; 58: 403–7. 10.1538/expanim.58.403 19654438

[185] Ngwenya LB , Heyworth NC, Shwe Y et al. Age-related changes in dentate gyrus cell numbers, neurogenesis, and associations with cognitive impairments in the rhesus monkey. Front Syst Neurosci 2015; 9: 102. 10.3389/fnsys.2015.00102 26236203 PMC4500920

[186] Sanai N , Nguyen T, Ihrie RA et al. Corridors of migrating neurons in the human brain and their decline during infancy. Nature 2011; 478: 382–6. 10.1038/nature10487 21964341 PMC3197903

[187] Bergmann O , Liebl J, Bernard S et al. The age of olfactory bulb neurons in humans. Neuron 2012; 74: 634–9. 10.1016/j.neuron.2012.03.030 22632721

[188] Ernst A , Alkass K, Bernard S et al. Neurogenesis in the striatum of the adult human brain. Cell 2014; 156: 1072–83. 10.1016/j.cell.2014.01.044 24561062

[189] Donega V , van der Geest AT, Sluijs JA et al. Single-cell profiling of human subventricular zone progenitors identifies SFRP1 as a target to re-activate progenitors. Nat Commun 2022; 13: 1036. 10.1038/s41467-022-28626-9 35210419 PMC8873234

[190] Dennis CV , Suh LS, Rodriguez ML et al. Human adult neurogenesis across the ages: an immunohistochemical study. Neuropathol Appl Neurobiol 2016; 42: 621–38. 10.1111/nan.12337 27424496 PMC5125837

[191] Yao J, Dai S and Zhu R et al. Deciphering molecular heterogeneity and dynamics of neural stem cells in human hippocampal development, aging, and injury. eLife 2023; 12: RP89507 https://elifesciences.org/reviewed-preprints/89507. 10.7554/eLife.89507 PMC11014727 38607670

[192] Ben Abdallah NM-B , Slomianka L, Vyssotski AL et al. Early age-related changes in adult hippocampal neurogenesis in C57 mice. Neurobiol Aging 2010; 31: 151–61. 10.1016/j.neurobiolaging.2008.03.002 18455269

[193] Tosoni G , Ayyildiz D, Bryois J et al. Mapping human adult hippocampal neurogenesis with single-cell transcriptomics: reconciling controversy or fueling the debate? Neuron 2023; 111:1714–31 https://www.cell.com/neuron/fulltext/S0896-6273(23)00206-4. 10.1016/j.neuron.2023.03.010 37015226

[194] Berg DA , Bond AM, Ming G-L et al. Radial glial cells in the adult dentate gyrus: what are they and where do they come from? F1000Res 2018; 7: 277. 10.12688/f1000research.12684.1 29568500 PMC5840617

[195] Brandt MD , Jessberger S, Steiner B et al. Transient calretinin expression defines early postmitotic step of neuronal differentiation in adult hippocampal neurogenesis of mice. Mol Cell Neurosci 2003; 24: 603–13. 10.1016/S1044-7431(03)00207-0 14664811

[196] Hodge RD , Kowalczyk TD, Wolf SA et al. Intermediate progenitors in adult hippocampal neurogenesis: Tbr2 expression and coordinate regulation of neuronal output. J Neurosci 2008; 28: 3707–17. 10.1523/JNEUROSCI.4280-07.2008 18385329 PMC6671086

[197] Berg DA , Su Y, Jimenez-Cyrus D et al. A common embryonic origin of stem cells drives developmental and adult neurogenesis. Cell 2019; 177: 654–68. 10.1016/j.cell.2019.02.010 30929900 PMC6496946

[198] Seki T , Hori T, Miyata H et al. Analysis of proliferating neuronal progenitors and immature neurons in the human hippocampus surgically removed from control and epileptic patients. Sci Rep 2019; 9: 18194. 10.1038/s41598-019-54684-z 31796832 PMC6890740

[199] Sorrells SF , Paredes MF, Velmeshev D et al. Immature excitatory neurons develop during adolescence in the human amygdala. Nat Commun 2019; 10: 2748. 10.1038/s41467-019-10765-1 31227709 PMC6588589

[200] Srikandarajah N , Martinian L, Sisodiya SM et al. Doublecortin expression in focal cortical dysplasia in epilepsy. Epilepsia 2009; 50: 2619–28. 10.1111/j.1528-1167.2009.02194.x 19583780

[201] Varea E, Castillo-Gómez E and Gómez-Climent MA et al. PSA-NCAM expression in the human prefrontal cortex. J Chem Neuroanat 2007; 33: 202–9. 10.1016/j.jchemneu.2007.03.006 17467233

[202] Verwer RWH , Sluiter AA, Balesar RA et al. Mature astrocytes in the adult human neocortex express the early neuronal marker doublecortin. Brain 2007; 130: 3321–35. 10.1093/brain/awm264 18055496

[203] Duque A , Arellano JI, Rakic P. An assessment of the existence of adult neurogenesis in humans and value of its rodent models for neuropsychiatric diseases. Mol Psychiatry 2022; 27: 377–82. 10.1038/s41380-021-01314-8 34667259 PMC8967762

[204] Duque A , Spector R. A balanced evaluation of the evidence for adult neurogenesis in humans: implication for neuropsychiatric disorders. Brain Struct Funct 2019; 224: 2281–95. 10.1007/s00429-019-01917-6 31278571 PMC6852840

[205] Lindvall O , Kokaia Z. Neurogenesis following stroke affecting the adult brain. Cold Spring Harb Perspect Biol 2015; 7: a019034. 10.1101/cshperspect.a019034 PMC4632663 26525150

[206] Jessberger S , Zhao C, Toni N et al. Seizure-associated, aberrant neurogenesis in adult rats characterized with retrovirus-mediated cell labeling. J Neurosci 2007; 27: 9400–7. 10.1523/JNEUROSCI.2002-07.2007 17728453 PMC6673128

[207] Parent JM , Elliott RC, Pleasure SJ et al. Aberrant seizure-induced neurogenesis in experimental temporal lobe epilepsy. Ann Neurol 2006; 59: 81–91. 10.1002/ana.20699 16261566

[208] Verret L , Jankowsky JL, Xu GM et al. Alzheimer's-type amyloidosis in transgenic mice impairs survival of newborn neurons derived from adult hippocampal neurogenesis. J Neurosci 2007; 27: 6771–80. 10.1523/JNEUROSCI.5564-06.2007 17581964 PMC4439193

[209] Winner B , Rockenstein E, Lie DC et al. Mutant alpha-synuclein exacerbates age-related decrease of neurogenesis. Neurobiol Aging 2008; 29: 913–25. 10.1016/j.neurobiolaging.2006.12.016 17275140 PMC2896275

[210] Li JY , Popovic N, Brundin P. The use of the R6 transgenic mouse models of Huntington's disease in attempts to develop novel therapeutic strategies. NeuroRx 2005; 2: 447–64. 10.1602/neurorx.2.3.447 16389308 PMC1144488

[211] Fedele V , Roybon L, Nordström U et al. Neurogenesis in the R6/2 mouse model of Huntington's disease is impaired at the level of NeuroD1. Neuroscience 2011; 173: 76–81. 10.1016/j.neuroscience.2010.08.022 20807561

[212] Croll L , Szabo CA, Abou-Madi N et al. Epilepsy in nonhuman primates. Epilepsia 2019; 60: 1526–38. 10.1111/epi.16089 31206636 PMC6779127

[213] Lin X , Wang H, Chen J et al. Nonhuman primate models of ischemic stroke and neurological evaluation after stroke. J Neurosci Methods 2022; 376: 109611. 10.1016/j.jneumeth.2022.109611 35487315

[214] Scott JT , Bourne JA. Modelling behaviors relevant to brain disorders in the nonhuman primate: are we there yet? Prog Neurobiol 2022; 208: 102183. 10.1016/j.pneurobio.2021.102183 34728308

[215] Beckman D , Ott S, Donis-Cox K et al. Oligomeric abeta in the monkey brain impacts synaptic integrity and induces accelerated cortical aging. Proc Natl Acad Sci USA 2019; 116: 26239–46. 10.1073/pnas.1902301116 31871145 PMC6936351

[216] Forny-Germano L , Lyra E Silva NM, Batista AF et al. Alzheimer's disease-like pathology induced by amyloid-beta oligomers in nonhuman primates. J Neurosci 2014; 34: 13629–43. 10.1523/JNEUROSCI.1353-14.2014 25297091 PMC6608380

[217] Yue F , Feng S, Lu C et al. Synthetic amyloid-βoligomers drive early pathological progression of Alzheimer's disease in nonhuman primates. iScience 2021; 24: 103207. 10.1016/j.isci.2021.103207 34704001 PMC8524197

[218] Sato K , Sasaguri H, Kumita W et al. A non-human primate model of familial Alzheimer's disease. Biorxiv 2020;2020.08.24.264259.

[219] Barth AL , Schneider JS, Johnston TH et al. NYX-458 improves cognitive performance in a primate Parkinson's Disease model. Mov Disord 2020; 35: 640–9. 10.1002/mds.27962 31967361

[220] Decamp E , Schneider JS. Attention and executive function deficits in chronic low-dose MPTP-treated non-human primates. Eur J Neurosci 2004; 20: 1371–8. 10.1111/j.1460-9568.2004.03586.x 15341609

[221] Li J , Li N, Wei J et al. Genetically engineered mesenchymal stem cells with dopamine synthesis for Parkinson's disease in animal models. NPJ Parkinsons Dis 2022; 8: 175. 10.1038/s41531-022-00440-6 36550118 PMC9780305

[222] Schneider JS , Kovelowski CJ 2nd. Chronic exposure to low doses of MPTP. I. Cognitive deficits in motor asymptomatic monkeys. Brain Res 1990; 519: 122–8. 10.1016/0006-8993(90)90069-N 2397401

[223] Schneider JS , Roeltgen DP. Delayed matching-to-sample, object retrieval, and discrimination reversal deficits in chronic low dose MPTP-treated monkeys. Brain Res 1993; 615: 351–4. 10.1016/0006-8993(93)90049-S 8364742

[224] Chan AWS , Jiang J, Chen Y et al. Progressive cognitive deficit, motor impairment and striatal pathology in a transgenic Huntington disease monkey model from infancy to adulthood. PLoS One 2015; 10: e0122335. 10.1371/journal.pone.0122335 25966278 PMC4428630

[225] Chan AWS , Xu Y, Jiang J et al. A two years longitudinal study of a transgenic Huntington disease monkey. BMC Neurosci 2014; 15: 36. 10.1186/1471-2202-15-36 24581271 PMC4015530

[226] Levchenko A , Kanapin A, Samsonova A et al. Human accelerated regions and other human-specific sequence variations in the context of evolution and their relevance for brain development. Genome Biol Evol 2018; 10: 166–88. 10.1093/gbe/evx240 29149249 PMC5767953

[227] Girskis KM , Stergachis AB, DeGennaro EM et al. Rewiring of human neurodevelopmental gene regulatory programs by human accelerated regions. Neuron 2021; 109: 3239–51. 10.1016/j.neuron.2021.08.005 34478631 PMC8542612

[228] Boyd JL , Skove SL, Rouanet JP et al. Human-chimpanzee differences in a *FZD8* enhancer alter cell-cycle dynamics in the developing neocortex. Curr Biol 2015; 25: 772–9. 10.1016/j.cub.2015.01.041 25702574 PMC4366288

[229] Bayne K , Hau J, Morris T. The welfare impact of regulations, policies, guidelines, and directives and nonhuman primate welfare. In: Robinson LM, Weiss A (eds.). Nonhuman Primate Welfare. Cham: Springer, 2023, 643–60.

[230] Feng G , Jensen FE, Greely HT et al. Opportunities and limitations of genetically modified nonhuman primate models for neuroscience research. Proc Natl Acad Sci USA, 2020 117: 24022–31. 10.1073/pnas.2006515117 PMC7533691 32817435

[231] Reillo I , De Juan Romero C, García-Cabezas MÁ et al. A role for intermediate radial glia in the tangential expansion of the mammalian cerebral cortex. Cereb Cortex 2011; 21: 1674–94. 10.1093/cercor/bhq238 21127018

[232] Hodge RD , Bakken TE, Miller JA et al. Conserved cell types with divergent features in human versus mouse cortex. Nature 2019; 573: 61–8. 10.1038/s41586-019-1506-7 31435019 PMC6919571

[233] Zhou Y , Bond AM, Shade JE et al. Autocrine Mfge8 signaling prevents developmental exhaustion of the adult neural stem cell pool. Cell Stem Cell 2018; 23: 444–52. 10.1016/j.stem.2018.08.005 30174295 PMC6128767

[234] Geschwind DH , Levitt P. Autism spectrum disorders: developmental disconnection syndromes. Curr Opin Neurobiol 2007; 17: 103–11. 10.1016/j.conb.2007.01.009 17275283

[235] Kana RK , Libero LE, Moore MS. Disrupted cortical connectivity theory as an explanatory model for autism spectrum disorders. Phys Life Rev 2011; 8: 410–37. 10.1016/j.plrev.2011.10.001 22018722

[236] Jung H , Park H, Choi Y et al. Sexually dimorphic behavior, neuronal activity, and gene expression in Chd8-mutant mice. Nat Neurosci 2018; 21: 1218–28. 10.1038/s41593-018-0208-z 30104731

[237] An Y , Zhang L, Liu W et al. De novo variants in the Helicase-C domain of CHD8 are associated with severe phenotypes including autism, language disability and overgrowth. Hum Genet 2020; 139: 499–512. 10.1007/s00439-020-02115-9 31980904

[238] Hoffmann A , Spengler D. Chromatin remodeler CHD8 in autism and brain development. J Clin Med 2021; 10: 366. 10.3390/jcm10020366 33477995 PMC7835889

[239] Hirotsune S , Fleck MW, Gambello MJ et al. Graded reduction of *Pafah1b1 (Lis1)* activity results in neuronal migration defects and early embryonic lethality. Nat Genet 1998; 19: 333–9. 10.1038/1221 9697693

[240] Reiner O , Sapir T. LIS1 functions in normal development and disease. Curr Opin Neurobiol 2013; 23: 951–6. 10.1016/j.conb.2013.08.001 23973156

[241] Khalaf-Nazzal R , Bruel-Jungerman E, Rio J-P et al. Organelle and cellular abnormalities associated with hippocampal heterotopia in neonatal *doublecortin* knockout mice. PLoS One 2013; 8: e72622. 10.1371/journal.pone.0072622 24023755 PMC3759370

[242] Lin J-R , Cheng J-F, Liu Y-T et al. Novel lissencephaly-associated *DCX* variants in the C-terminal DCX domain affect microtubule binding and dynamics. Epilepsia 2022; 63: 1253–65. 10.1111/epi.17198 35213059

